# Divergent evolution of Di-lysine ER retention vs. farnesylation motif-mediated anchoring of the AnkB virulence effector to the *Legionella*-containing vacuolar membrane

**DOI:** 10.1038/s41598-017-05211-5

**Published:** 2017-07-11

**Authors:** John D. Perpich, Awdhesh Kalia, Christopher T. D. Price, Snake C. Jones, Kathy Wong, Kalle Gehring, Yousef Abu Kwaik

**Affiliations:** 1 Department of Microbiology and Immunology, College of Medicine, University of Louisville, Louisville, KY USA; 2 Graduate Program in Diagnostic Genetics, School of Health Professions, The University of Texas MD Anderson Cancer Center, Houston, TX USA; 3 Department of Biochemistry and Groupe de recherche axé sur la structure des protéines, McGill University, Montreal, Quebec H3G 0B1 Canada; 4 Center for Predictive Medicine, University of Louisville, Louisville, KY USA

## Abstract

Within macrophages and amoeba, the *Legionella*-containing vacuole (LCV) membrane is derived from the ER. The *bona fide* F-box AnkB effector protein of *L*. *pneumophila* strain AA100/130b is anchored to the cytosolic side of the LCV membrane through host-mediated farnesylation of its C-terminal eukaryotic “CaaX” motif. Here we show that the AnkB homologue of the Paris strain has a frame shift mutation that led to a loss of the CaaX motif and a concurrent generation of a unique C-terminal KNKYAP motif, which resembles the eukaryotic di-lysine ER-retention motif (KxKxx). Our phylogenetic analyses indicate that environmental isolates of *L*. *pneumophila* have a potential positive selection for the ER-retention KNKYAP motif. The AnkB-Paris effector is localized to the LCV membrane most likely through the ER-retention motif. Its ectopic expression in HEK293T cells localizes it to the perinuclear ER region and it *trans-*rescues the *ankB* mutant of strain AA100/130b in intra-vacuolar replication. The di-lysine ER retention motif of AnkB-Paris is indispensable for function; most likely as an ER retention motif that enables anchoring to the ER-derived LCV membrane. Our findings show divergent evolution of the *ankB* allele in exploiting either host farnesylation or the ER retention motif to be anchored into the LCV membrane.

## Introduction

*Legionella pneumophila* is an environmental organism that proliferates within various protists hosts in the aquatic environment^1–3^. Co-evolution and adaptation of *L*. *pneumophila* to the intracellular lifestyle within protists is believed to have played a major role in its ability to exploit evolutionarily conserved eukaryotic processes that enables its proliferation within human cells^1–3^. Upon aerosol transmission from the aquatic environment to humans as planktonic or in biofilms^4^, *L*. *pneumophila* replicates within alveolar macrophages, causing Legionnaires’ disease^5^.

Upon attachment to macrophages and amoeba through pili and other attachment factors^6–8^, the bacteria are internalized. Within both evolutionarily distant host cells, the *L*. *pneumophila-*containing vacuole (LCV)^9^ evades endocytic fusion and intercepts ER-to-Golgi vesicle traffic to be remodeled into an ER-derived vacuole^10–15^. The Dot/Icm type IV secretion system^16, 17^ injects into the host cell a cadre of ~300 effectors^18, 19^ to modulate a myriad of cellular processes involved in biogenesis of the LCV and to re-program the host cell into a proliferation niche^11, 12, 20–23^. Most of the effectors are dispensable for intracellular proliferation of *L*. *pneumophila* ^24^. The Ankyrin B (AnkB) effector of *L*. *pneumophila* is one of very few effectors required for the intracellular proliferation and exploits the evolutionarily-conserved ubiquitin-proteasome machinery within mammalian and protozoan cells^25, 26^. The crystal structure of the AnkB effector shows that it is composed of three ankyrin repeats and an F-box domain^27^.

Anchored to the LCV membrane, the *bona fide* F-box AnkB effector interacts with the host SCF1 ubiquitin ligase complex^25, 26, 28–30^. On the LCV membrane, AnkB functions as a platform for the docking of Lys^48^- linked polyubiquitinated proteins to the LCV membrane within human cells and amoeba as well as *Drosophila*-derived cells, and the process is initiated upon bacterial attachment to the plasma membrane^25, 26, 29, 31, 32^. High throughput proteomic analyses of the LCV polyubiquitinated proteome have identified several host proteins specifically polyubiquitinated in an AnkB-dependent manner^33^. The AnkB-assembled Lys^48^ -linked polyubiquitinated proteins are targeted to proteasomal degradation^34^, which is essential to raise the concentration of host cell amino acids above the threshold needed as major sources of carbon and energy for the robust intra-vacuolar proliferation of *L*. *pneumophila* within mammalian and protozoan cells^5, 29, 35, 36^. Thus, AnkB is designated as a nutritional virulence effector^37–41^. Interestingly, AnkB has been recently shown to be to bind the Trim 21 host ubiquitin ligase and to become ubiquitinated by K^11^- linked polyubiquitination^32^, but the role of this ubiquitination is AnkB function is not known.

Post-translational modification of hydrophilic eukaryotic proteins through farnesylation is mediated by the covalent addition of a 15-carbon farnesyl isoprenoid lipid moiety that enables anchoring of the farnesylated proteins to the lipid bi-layer of eukaryotic membranes^36, 42, 43^. Anchoring of the AnkB effector of strain AA100/130b of *L*. *pneumophila* into the LCV membrane is mediated through host-mediated farnesylation of the cysteine residue at the -4 position from the C-terminus within the eukaryotic-like “**C**aaX” motif^3, 36, 44^. Similar to genetic ablation of *ankB* ^25, 26, 45^, substitution of the cysteine residue within the CaaX motif results in a total loss of biological function of AnkB of strain AA100/130b, which leads to a defect in intracellular proliferation within amoeba and human cells and in attenuation in the mouse model of Legionnaires disease^44^. In addition to farnesylation, AnkB has been recently shown to be modified through asparagine hydroxylation by the host asaparaginyl hydroxylase, which is recruited to the LCV in a Dot/Icm-dependent manner^46^.

Similar to strain AA100/130b, the AnkB homologue of strain Paris (AnkB-Paris) mediates decoration of the LCV with polyubiquitinated proteins, and is also required for intracellular proliferation and for virulence in the mouse model, yet to a lesser extent than strain AA100/130b^26, 45^. Surprisingly, while the C-terminal CaaX motif of AnkB-AA100/130b is indispensable for anchoring the effector to the LCV membrane through host-mediated farnesylation, which is essential for function, the CaaX motif is absent from AnkB-Paris. Ectopically expressed AnkB-AA100/130b in HEK293 cells or amoeba becomes farnesylated and is uniformly localized throughout the cytosolic side of the plasma membrane where it recruits polyubiquitinated proteins that are degraded by the proteasomes^25, 36, 44^. Importantly, farnesylation-mediated anchoring of AnkB-AA100/130b into the plasma membrane is essential for *trans*-rescue of the *ankB* mutant in intracellular proliferation^25^. In contrast to AnkB-AA100/130b, upon ectopic expression within A549 cells, AnkB-Paris is enriched at the leading edge of lamellipodium formation and co-localizes with α-actinin^26^. However, sub-cellular location of AnkB-Paris during infection is not known. Compared to the *ankB*-AA100/130b allele, there is a single nucleotide deletion in the *ankB*-*Paris* leading to a frame shift, which results in a truncation of the protein for the last 18 amino acids residues, which include a portion of the third ankyrin repeat domain and the CaaX farnesylation motif. However, the respective frame shift in AnkB-Paris generated a unique C-terminus, which resembles a eukaryotic di-lysine ER-retention motif (KxKxx)^47^. The crystal structure of AnkB-AA100/130b indicates that the C-terminal truncation of AnkB-Paris eliminates a large portion of the third ankyrin repeat compared to AnkB-AA100/130b^48^. Importantly, the AnkB lysine residues modified by K^11^-linked polyubiquitination are conserved in the two AnkB effectors^32^.

Here we show that among 51 unique clinical and environmental isolates there is predominance and selection of the *ankB*-Paris allele in environmental isolates. The divergent evolution of *ankB*-*Paris* allele has led to acquisition of a C-terminal putative di-lysine ER retention motif, which is indispensable for biological function. The di-lysine ER retention motif likely enables anchoring of AnkB-Paris to the ER-derived LCV membrane, in contrast to the CaaX motif farnesylation-mediated anchoring of AnkB-AA100/130b. Despite truncation of the third ankyrin repeat domain in AnkB-Paris, it can functionally substitute for AnkB-AA100/130b strain in decoration of the LCV with polyubiquitinated proteins for rescue of intra-vacuolar proliferation.

## Results

### Episodic Positive Selection in *ankB* Evolution

**Figure 1:**
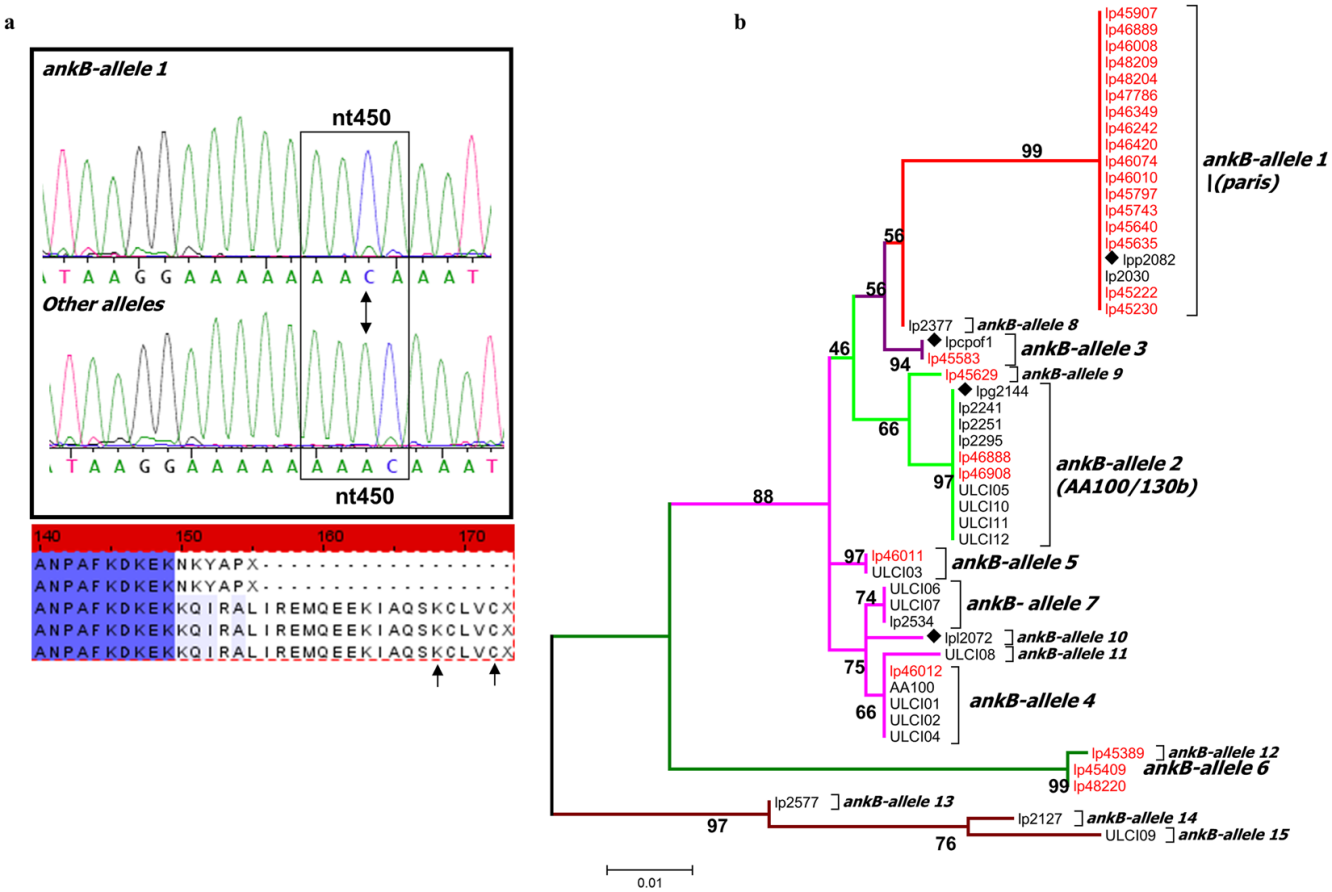
Molecular evolution of *ankB*. (**a**) Representative sequence chromatograms indicating the frame shift mutation at nucleotide position 450 (*ΔA*) in *ankB1* compared to other *ankB* alleles. This mutation in *ankB*1 alters the reading frame and predicts a prematurely terminated AnkB protein at residue 154 and generation of a unique NKYAP sequence motif. Arrows mark the location of the Dot-Icm translocation signal. (**b**) ML analysis of *ankB* alleles variously from clinical (in black text) and environmental (in red text) isolates. All alleles are indicated. Diamonds indicate *ankB* sequence from strains whose complete genomes has been determined. Bootstrap values are shown above the branches. The tree is drawn to scale, with branch lengths measured in the number of substitutions per site.

Compared to strain AA100/130b, the *ankB* gene of the Paris strain (*ankB*-Paris) has a deletion of an adenine at position 450 (ΔA450), which resulted in a frame shift mutation (Fig. 1). This has led to a truncation of the last 18 amino acids that included the CaaX farnesylation motif, which is essential for anchoring AnkB-AA100/130b into the LCV membrane, which is indispensable for its biologic function in decorating the LCV with polyubiquitinated proteins^36, 44^. Despite this frame shift mutation and deletion of the C-terminal CaaX farnesylation motif, AnkB-Paris is required for decoration of the LCV with polyubiquitinated proteins^26^. Concurrently, a unique NKYAP sequence motif is generated at amino acids 150–154 in AnkB-Paris. To determine whether this frame shift mutation was unique to the Paris strain or more widespread among other *Legionella* isolates, we examined the abundance of the ΔA450 mutation in fifty-one isolates of clinical (N = 25) and environmental (N = 26) origin. Analysis of full-length *ankB* sequences revealed 15 distinct *ankB* alleles (Fig. 1b; Fig. S1). Interestingly, among the 15 *ankB* alleles, *ankB1* (Paris strain) was the only *ankB* allele to harbor the ΔA450 mutation (Fig. 1b). The data showed that 19 of 51 isolates (37.25%), of which 17 were environmental and 2 were of clinical origin, contained *ankB1* allele (*ankB1*/*ankB-Paris*). We conclude that the *ankB1*/*ankB-Paris* allele is wide-spread and is predominant among environmental isolates.

To better understand the forces that shaped *ankB* evolution, in particular the maintenance and spread of the *ankB1* allele in environmental *Legionella* isolates, we next analyzed the selective pressures acting on *ankB* codons and also on the *ankB1* branch using CODEML and a variety of other methods (materials and methods). We found preponderance of sites that were constrained by either negative selection or evolved neutrally. Comparisons of models M7 and M8 also suggested that at least 4 *ankB* codons had been subject to positive selection (Table S1). However, we also found evidence of recombination in the *ankB* alignment (Fig. S2, Table S2). Although the M7 vs M8 comparison should be robust to model violations introduced due to recombination, we sought additional evidence to verify positive selection using REL, FEL, IFEL and MEME methods (materials and methods). Each of these methods found significant statistical support for site-specific positive selection in *ankB* codons (Tables S3–S7). Branch site tests implemented in CODEML, and the GA branch test both provided statistical support for the hypotheses that the internal branch leading up to *ankB1*/*ankB-Paris* and its branch both had experienced positive selection. Moreover, the NKYAP C-terminal motif itself was identified as the target of positive selection (H S1; Fig. S3 and Table S7).

### Decoration of the LCV with Polyubiquitinated Proteins Independent of the *ankB* Genotype

**Figure 2:**
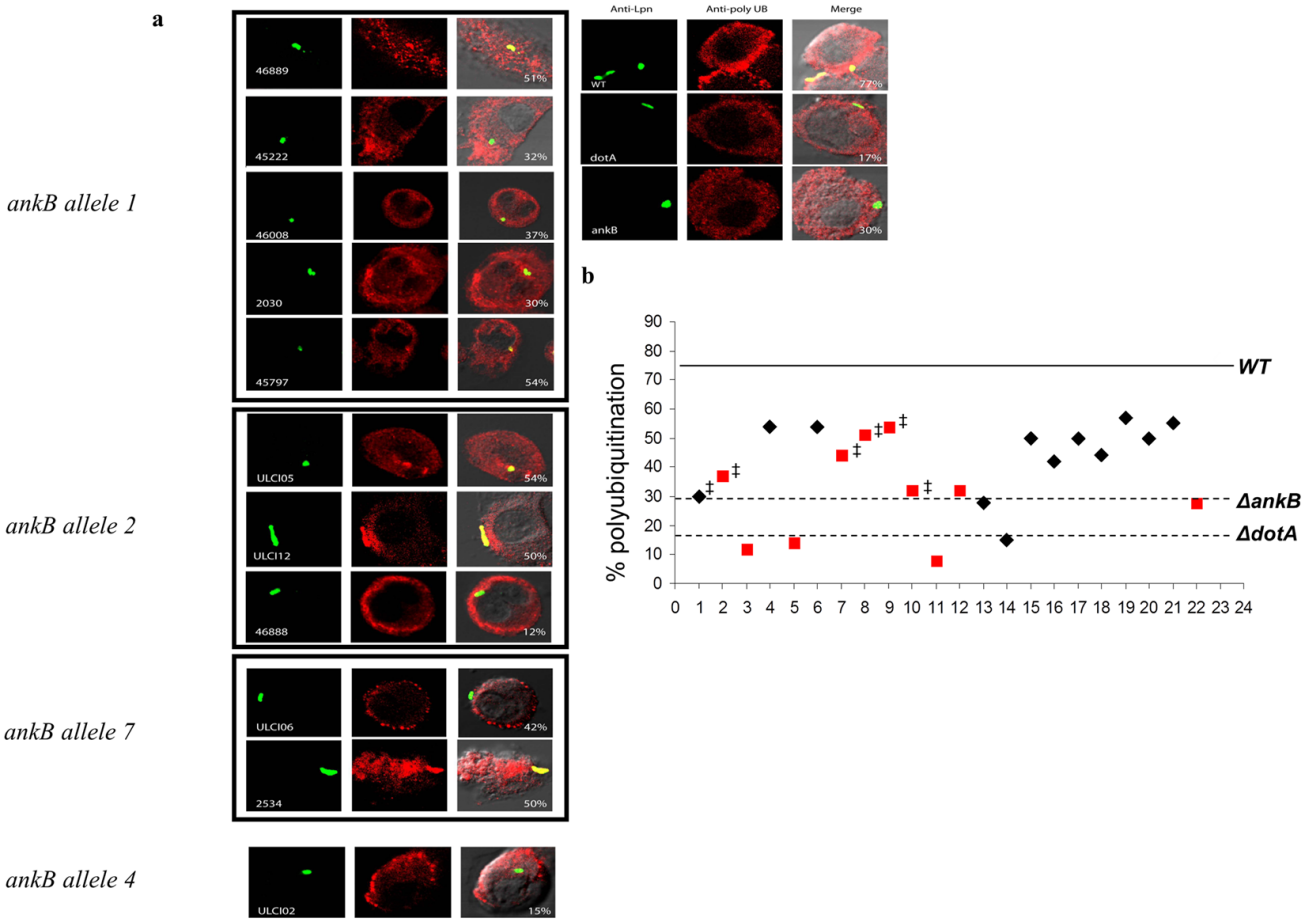
AnkB-genotype does not predict the ability to recruit polyubiquitinated proteins to the LCV. (**a**) Representative confocal microscopy images of polyubiquitinated protein recruitment to the LCV among 23 different isolates of *L*. *pneumophila* expressing various *ankB* alleles. Percentages indicate the number of LCVs positive for ubiquitin. Images in the right panel were taken using the AA100 strain. (**b**) Distribution of percent polyubiquitin recruitment among 23 different isolates of *L*. *pneumophila*. Environmental isolates are shown in red and clinical isolates are shown in black. ^‡^, indicates strains carrying the *ankB1* allele.

We next asked whether the altered C-terminus of AnkB variant encoded by *ankB*1 (Fig. 1a) either modified or significantly reduced the ability of AnkB1 strains to recruit polyubiquitinated proteins to the LCV. Overall, we found extensive variation in the ability of 23 distinct *Legionella* isolates to recruit polyubiquitinated proteins to the LCV (Fig. 2a and b). Specifically, among AnkB1 strains, polyubiquitination varied from 30% to 54%. In contrast, six isolates with full-length *ankB* alleles were either similar to the Δ*ankB* mutant or the *ΔdotA* translocation-defective mutant in their ability to recruit polyubiquitinated proteins to the LCV (Fig. 2a and b). However, a comparison of environmental and clinical isolates revealed a modest, but statistically significant difference (Student *t*-test, P < 0.05), in their ability to recruit polyubiquitinated proteins (Fig. S4). Thus, while the environmental isolates seem less capable than clinical isolates to recruit polyubiquitinated proteins to the LCV, the ability to recruit polyubiquitinated proteins itself seems independent of the *ankB* genotype.

### Localization of AnkB-Paris to the LCV membrane

**Figure 3:**
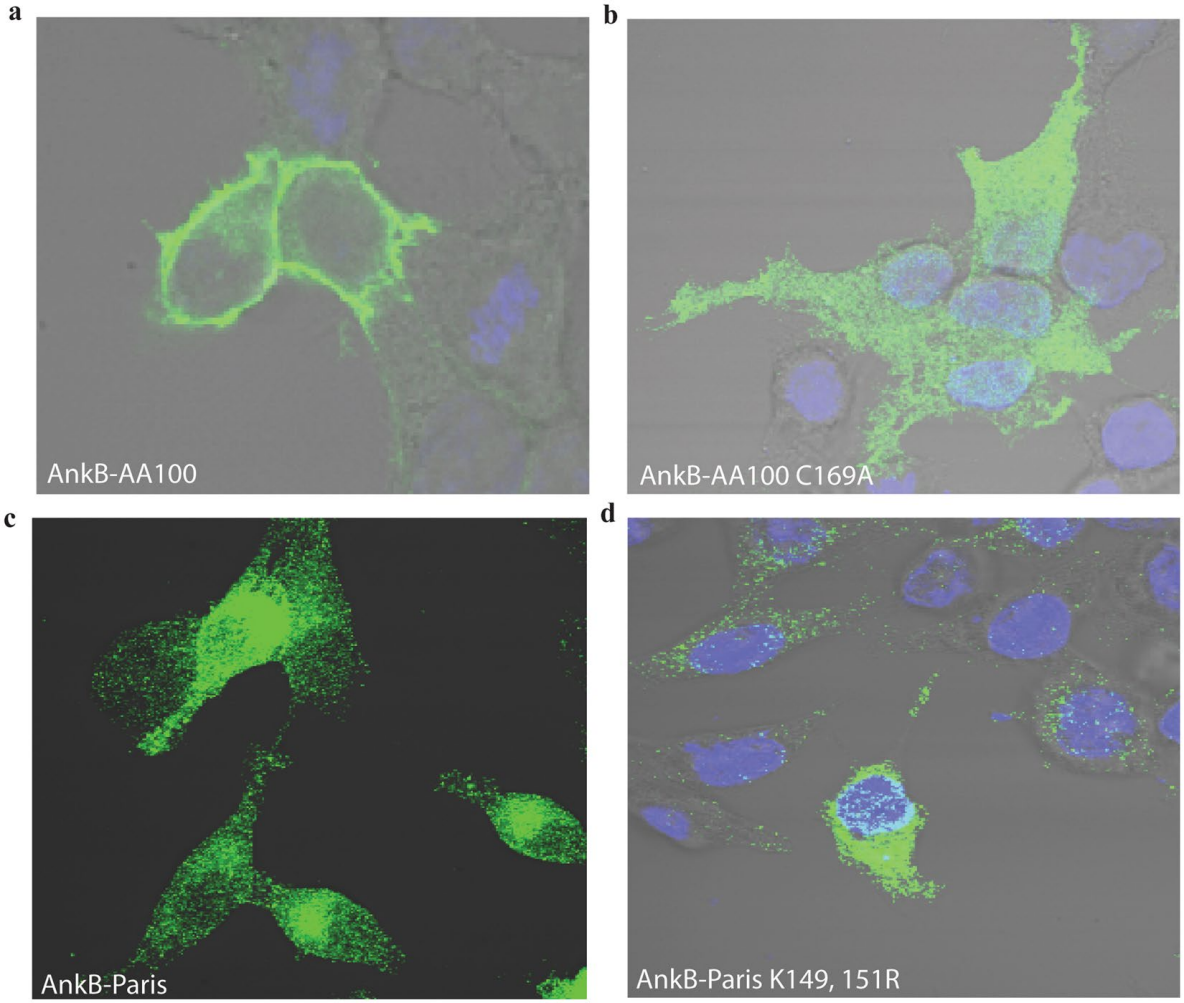
Ectopically expressed AnkB-Paris localizes to the cytoplasm with a perinuclear distribution. (**a**–**d**) Localization of AnkB-AA100, AnkB-Paris, AnkB-Paris K^149,151^R, and AnkB-C169A in HEK293T cells transiently transfected with 3X Flag-tagged versions of each and stained with anti-flag antibodies and DAPI. Representative confocal images are shown.

Ectopic expression of AnkB-AA100/130b within amoeba and HEK293T cells results in farnesylation-mediated anchoring to the plasma membrane of both evolutionarily distinct host cells^36, 44, 49, 50^. In contrast to AnkB-AA100/130b, AnkB-Paris ectopically expressed within A549 cells is enriched at the leading edge of lamellipodium formation and co-localizes with α-actinin^26^. Our data showed that in contrast to AnkB-AA100/130b (Fig. 3a), when AnkB-Paris is ectopically expressed in HEK293T cells, it did not localize to the plasma membrane; but it exhibited a punctate appearance and a perinuclear distribution, which is characteristic of sub-cellular localization of the ER (Fig. 3c). This perinuclear distribution is distinct from the diffuse cytosolic pattern characteristic of AnkB-C169A, which lacks the farnesylation motif (Fig. 3b). Interestingly, mutation of both lysines within the di-lysine motif of AnkB-Paris to arginine (AnkB-Paris K^149,151R^) did not grossly alter the distribution upon ectopic expression (Fig. 3d). In addition, labeling cells for co-localization with calnexin failed to show that either AnkB-Paris or AnkB-Paris^K149,151R^ localized to the ER (data not shown). Therefore, ectopically expressed AnkB-Paris was localized to the perinuclear ER region while AnkB-Paris^K149,151R^ was distributed throughout the cytoplasm, suggesting the loss of ER localization mediated by the ER retention motif (Fig. 3). Our data clearly show a distinct sub-cellular localization of AnkB-Paris and AnkB-AA100/130b.

**Figure 4:**
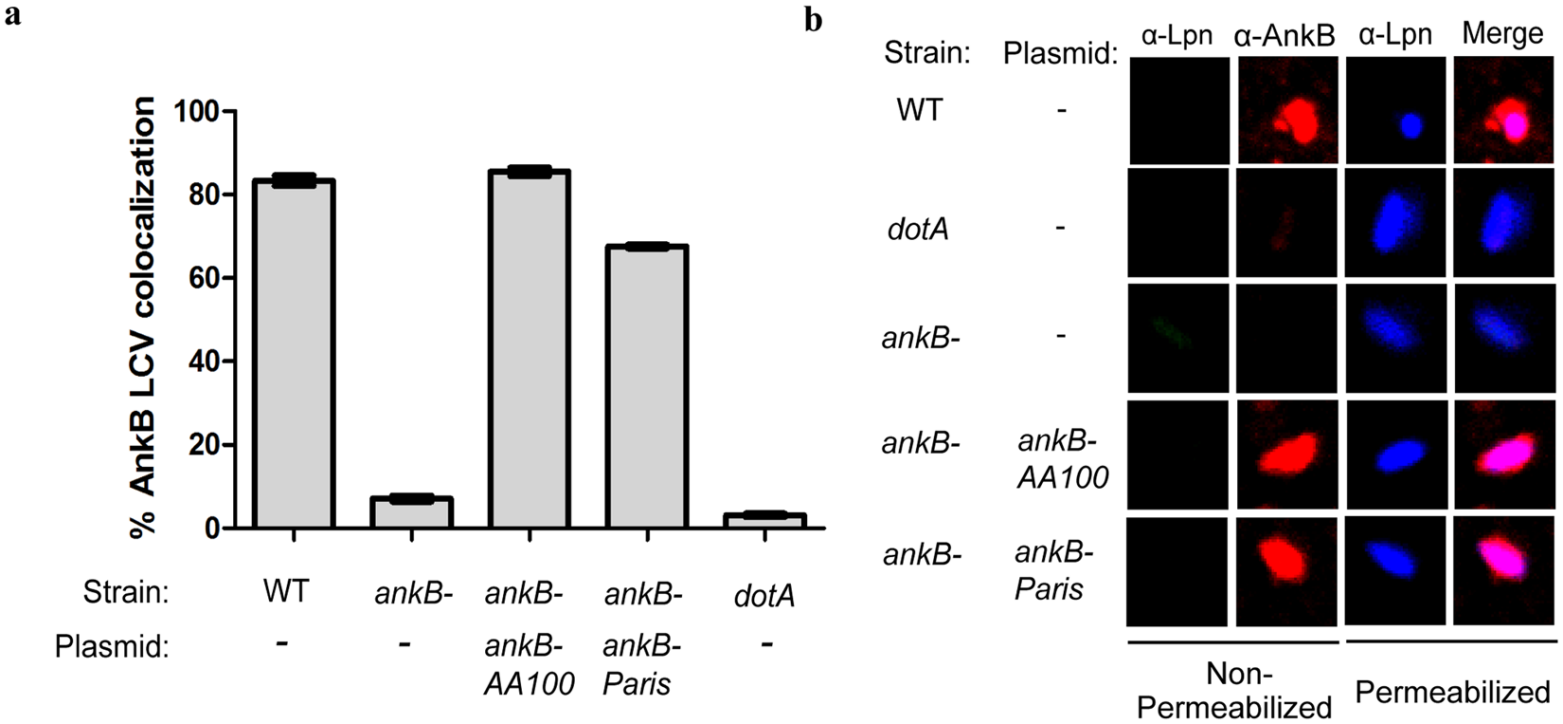
AnkB-Paris localizes to the LCV during infection. (**a**) Macrophages infected with WT *L*. *pneumophila* AA100/130b, *ankB* mutant, or the *ankB* mutant complemented with either *ankB* from AA100/130b or *ankB* from Paris strain were fixed at 2 hours post-infection and stained with antibodies to AnkB and *Legionella* (Lpn). The percentage of bacteria staining positive for AnkB (mean ± 1 SD) was determined by analysis of 100 infected cells in triplicate. Data are representative of 2 independent experiments. (**b**) Representative confocal images of LCVs isolated from macrophages infected with the indicated strains. To differentiate between intact LCVs and extracellular bacteria, the LCVs were labeled prior to permeabilization with mouse anti*-*Lpn antisera and rabbit anti-AnkB antisera for 1 h. LCVs were then permeabilized with −20 °C methanol and counter-labeled with goat anti-Lpn antisera to detect intact LCVs. Abbreviations: *ankB-* (*ankB* null mutant in AA100/130b strain), *dotA* (*dotA* null mutant in AA100/130b strain), WT (wild type AA100/130b strain). Plasmids indicate the *ankB* allele used to complement the indicated strain.

While AnkB-AA100/130b is localized to the LCV membrane during infection by host-mediated farnesylation, sub-cellular location of AnkB-Paris during infection is not known. Since the farnesylation motif is missing from AnkB-Paris, we set out to determine sub-cellular localization of AnkB-Paris during infection of hMDMs. We created an identical *ankB-Paris* allele and introduced it into the isogenic *ankB* null mutant of strain AA100/130b to determine its potential anchoring to the LCV membrane despite the lack of the farnesylation motif. At 2 hours post-infection, the LCVs were semi-purified from infected hMDMs. Prior to their permeabilization, the LCVs were labeled with anti-AnkB antibodies to detect AnkB on the cytosolic side of the LCV membrane, as we described previously^44^. Analyses by confocal microscopy showed that AnkB-AA100/130b was anchored to the cytosolic side of the LCV membrane of 80% of WT strain-containing LCVs (Fig. 4a and b). As expected, complementation of the *ankB* mutant of strain AA100/130b with *ankB*-AA100/130b restored localization of AnkB to the LCV membrane similar to the wild type strain^44^. Interestingly, despite the lack of the farnesylation motif, complementation of the AA100/130b-derived *ankB* mutant with the *ankB-Paris* allele resulted in anchoring AnkB-Paris to the LCV membrane, similar to AnkB-AA100/130b. Therefore, despite lacking the farnesylation motif, which is indispensable for anchoring AnkB-AA00/130b to the ER-derived LCV membrane, AnkB-Paris is also anchored to the LCV membrane, and this is likely to be mediated the di-lysine ER retention motif.

### Functional substitution of AnkB-AA100/130b by AnkB-Paris

**Figure 5:**
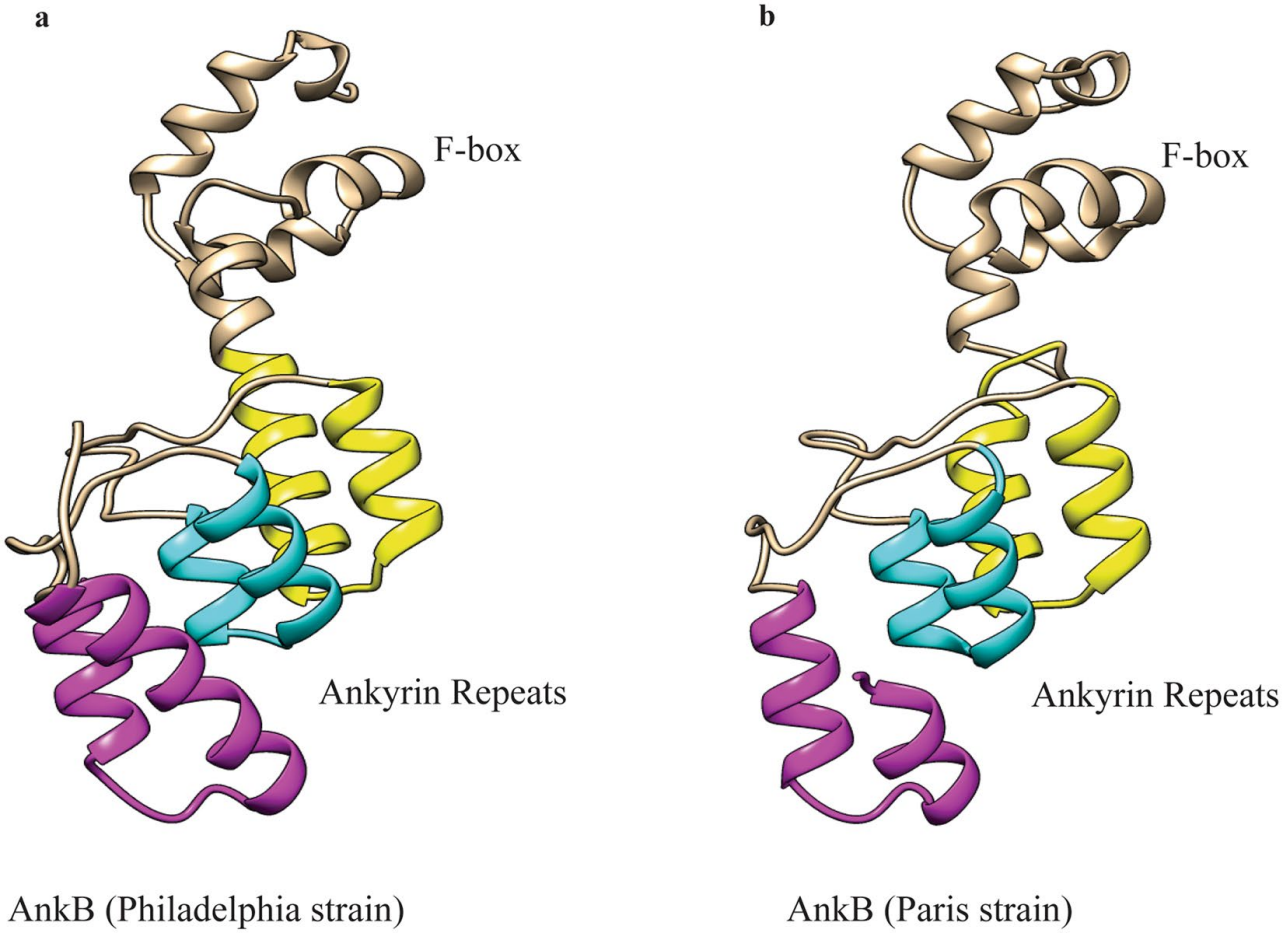
The crystal structure of AnkB. (**a**) The structure of AnkB from the Philadelphia strain spanning from Lys2 to Ala168^48^. The F-box domain near the N-terminus is indicated. Three ankyrin repeats are present rather than the predicted two repeats based on the sequence (yellow, cyan, and magenta). (**b**) Predicted structure of AnkB-Paris, which maintains all three ankyrin repeats by keeping the last half of the last repeat (magenta).

Compared to AnkB-AA100/130b, the AnkB-Paris has a truncation of the last 18 amino acids. The crystal structure of AnkB indicates that the C-terminal truncation of AnkB-Paris eliminates a large portion of the third ankyrin repeat compared to AnkB-AA100/130b (Fig. 5)^48^. To determine if AnkB-Paris can functionally substitute for AnkB-AA100/130b strain, we complemented the *ankB* null mutant of the AA100/130b strain with the *ankB-Paris* allele and assessed intracellular replication and decoration of the LCV with polyubiquitinated proteins within human monocytes-derived macrophages (hMDMs)^36, 44^.

**Figure 6:**
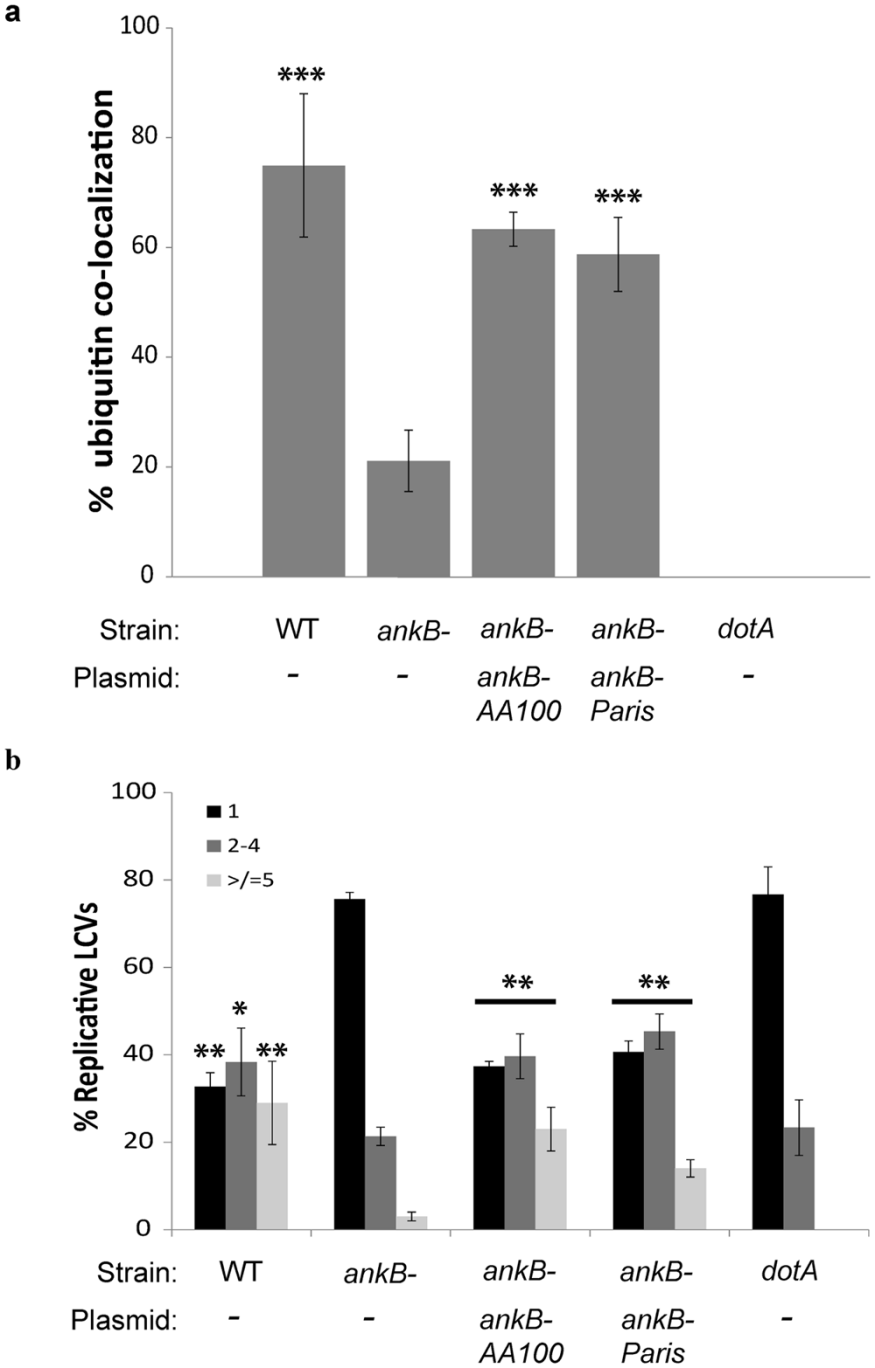
AnkB-Paris complements the *ankB* mutant of strain AA100/130b. (**a**) Co-localization of polyubiquitinated proteins with the LCV at 2 hours post-infection of hMDMs. Macrophages were infected with either wild type *L*. *pneumophila* strain AA100/130b, its isogenic *ankB* or *dotA* mutant, or the *ankB* mutant complemented with *ankB-Paris* or *ankB-AA100*. Numbers indicate the percentage of LCVs ( ± 1 SD) that co-localize with polyubiquitinated proteins. The data are based on analysis of 100 infected cells performed in triplicate and are representative of three independent experiments. (**b**) At 10 hours post-infection hMDMs were fixed, stained with anti-Lpn, and analyzed by confocal microscopy. The number of bacteria per cell was determined and the data are based on analysis of 100 infected cells (mean ± 1 SD) performed in triplicate and are representative of three independent experiments. *p < 0.05, **p < 0.01, ***p < 0.001 compared to corresponding value for *ankB* null mutant.

We assessed polyubiquitination of the vacuole at 2 hours post-infection by confocal microscopy. The data showed that 20% of *ankB* mutant-containing LCVs were decorated with polyubiquitinated proteins (Fig. 6a). In contrast, approximately 75% of strain AA100/130b-containing LCVs were decorated with polyubiquitinated proteins. Despite truncation of the third ankyrin domain, the *ankB-Paris* allele fully complemented the AA100/130b isogenic *ankB* null mutant for accumulation of polyubiquitinated proteins, similar to the wild type strain.

We assessed the ability of AnkB-Paris to restore intracellular replication of the *ankB* mutant of strain AA100/130b by determination of the frequency of formation of replicative vacuoles at 10 hours post-infection, by confocal microscopy (Fig. 6b). The majority of cells infected with the *ankB* mutant contained a single bacterium. In contrast, the majority of cells infected with WT bacteria contained 2–4 bacteria per cell and >20% of the LCVs harbored more than 5 bacteria per cell. Similarly, the *ankB* mutant complemented with the *ankB-Paris* allele formed replicative vacuoles at a frequency similar to the WT strain (Fig. 6b). Taken together, these results indicate that AnkB-Paris is functionally equivalent to AnkB-AA100/130b in its ability to decorate the LCV with polyubiquitinated proteins and to power intracellular proliferation of *L*. *pneumophila*.

### An indispensable role for the C-terminal Di-lysine Motif of AnkB-Paris in biological function

**Figure 7:**
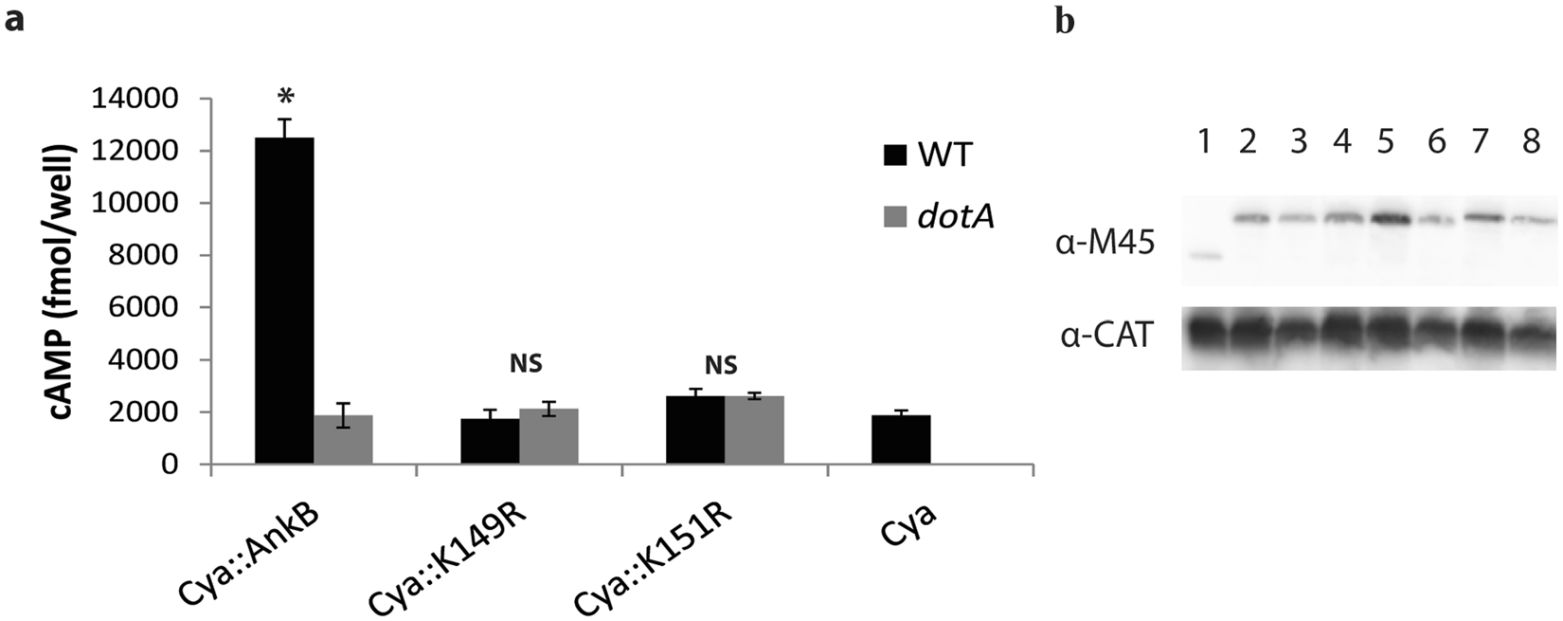
The putative di-lysine motif in the C-terminus of AnkB-Paris is essential for translocation by the Dot/Icm system. (**a**) Translocation of AnkB-Paris into U937 cells at 2 hours post-infection by WT or *dotA* mutant bacteria expressing either Cya (negative control) or the indicated Cya::AnkB-Paris fusions. Data represent the mean cAMP concentration of 3 wells (±1 SD). *p < 0.005 compared to *dotA* harboring Cya::AnkB-Paris. (**b**) Proteins derived from 1 × 10^8^ bacteria were loaded onto an SDS-PAGE gel and expression of fusion constructs was detected by Western blot using an antibody to the M45 epitope present in all Cya fusions. Blots were re-probed with anti-CAT antibodies. Lanes 1: WT Cya, 2: *dotA* Cya-AnkB-Paris K^151^R, 3: WT Cya-AnkB-Paris K^151^R, 4: *dotA* Cya-AnkB-Paris K^151^R, 5: *dotA* Cya-AnkB-Paris K^149^R, 6: WT Cya-AnkB-Paris K^149^R, 7: *dotA* Cya-AnkB-Paris, 8: WT Cya-AnkB-Paris.

The C-terminal 5 residues of AnkB strain Paris (K^149^NK^151^YAP) resemble a eukaryotic di-lysine motif (KxKxx) responsible for ER-to-golgi retrograde protein trafficking and retention in the ER^51–54^. The AnkB-AA100/130b is anchored to the LCV membrane by host-mediated farnesylation, which is essential for biological function. Therefore, we tested the hypotheses that the generated di-lysine ER-retention motif is also required for biological function of AnkB-Paris. We constructed single and double substitutions of lysine^149^ and lysine^151^ in the *ankB-Paris* allele with arginine. Since *L*. *pneumophila* effectors often have Dot/Icm translocation signals encoded in their C-terminus, we tested the ER retention motif AnkB-Paris^K149R^ and AnkB-Paris^K151R^ substitution mutants^45^ for Dot/Icm-mediated translocation using an adenylate cyclase reporter assay, as we described previously^25, 44, 45^. The *cya* reporter fusions of *ankB-Paris*, *ankB-Paris* ^K149R^, or *ankB-Paris* ^K151R^ were transformed into either the WT strain AA100/130b or its isogenic translocation-deficient *dotA* mutant. After 2 hours of infection, cells were lysed and cAMP levels were determined via ELISA (Fig. 7a and b). Cells infected with WT bacteria expressing the Cya-AnkB-Paris reporter fusion showed robust cAMP production compared to cells infected with *dotA* mutant bacteria expressing the same reporter fusion or cells infected with the WT strain expressing the catalytic domain of Cya alone. This indicates that AnkB-Paris is translocated by the AA100/130b strain. In contrast, substitution of either K^149^ or K^151^ completely abolished translocation of AnkB-Paris. These results indicate that the two lysine residues in the putative di-lysine ER-retention motif are essential for translocation of AnkB-Paris during infection.

In eukaryotic cells, the di-lysine motif is recognized by the coatomer complex (COPI). Coatomer is a multiprotein complex composed of two subcomplexes that include a trimer of α-COP, β’-COP, and ε-COP and a tetramer composed of β-COP, γ-COP, δ-COP, and ζ-COP^55^. The α-COP and β’-COP subunits of coatomer are responsible for binding di-lysine motifs. We tested for a physical interaction between AnkB-Paris and α-COP or β’-COP *in vivo* by Co-IP but were unable to detect any interaction. It is possible that overexpression of multiple members of the COPI complex is required to detect a physical interaction with AnkB-Paris.

### The Putative Di-lysine Motif of AnkB-Paris is Required for *in-trans* Rescue of the *ankB* Mutant

**Figure 8:**
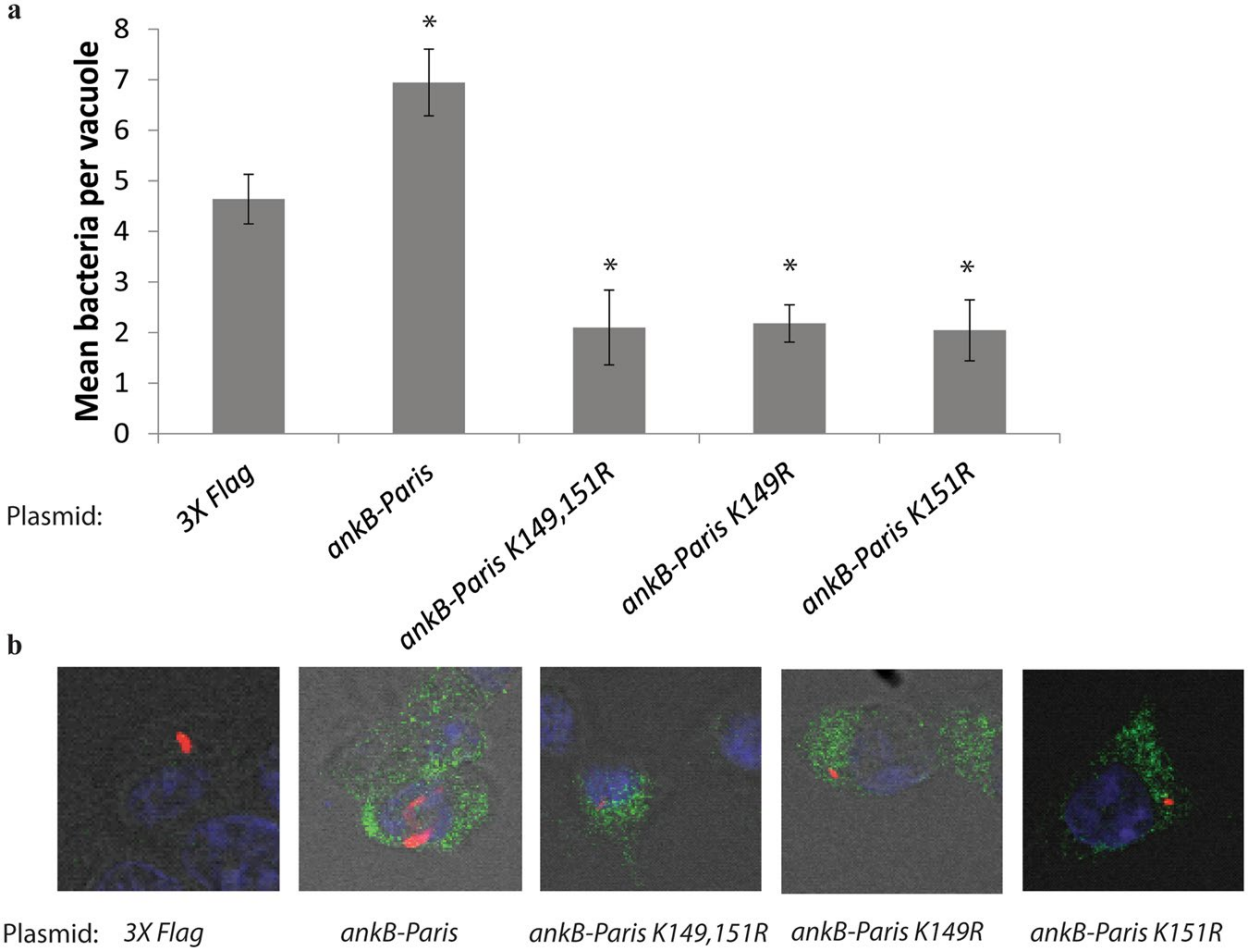
Requirement of the putative di-lysine motif in the C-terminus of ectopically expressed AnkB-Paris for *trans*-rescue of the *ankB* mutant. (**a**) HEK293T cells were first transfected with plasmids encoding 3X-Flag empty vector, 3X-Flag AnkB-Paris, 3X-Flag AnkB-Paris K^149,151^R, 3X-Flag AnkB-Paris K^149^R, or 3X-Flag AnkB-Paris K^151^R and then infected with the *ankB* mutant. Intracellular replication was analyzed at 10 hours post infection by confocal microscopy. The results are based on examination of 50 infected/transfected cells using three biological replicates. The mean number of bacteria per transfected HEK293T cell is shown. Error bars represent 1 standard deviation. *p < 0.01 compared to cells transfected with empty vector. (**b**) Representative confocal microscopy images of cells infected with the *ankB* mutant and expressing the indicated *3X Flag-ankB* fusion. Anti-flag staining is shown in green and anti-Lpn is shown in red.

Since AnkB-Paris^K149R^ and AnkB-Paris^K151R^ are not translocated by the Dot/Icm system, we could not test the potential effect of these mutations on intracellular growth or decoration of the LCV with polyubiquitinated proteins. We have previously shown that the *ankB* null mutant of strain AA100/130b is rescued for intra-vacuolar growth within HEK293T ectopically expressing AnkB but not by the farnesylation-defective AnkB variant that has a substitution of the cysteine within the C-terminal CaaX farnesylation motif^44^. This is due to the ability of ectopically expressed AnkB to be farnesylated and anchored to the cytosolic side of the plasma membrane where polyubiquitinated proteins are assembled, while the farnesylation defective variant of AnkB is defective. Since this approach bypasses the need for translocation, we transfected 3X Flag-tagged versions of AnkB-Paris, AnkB-Paris^K149R^ and AnkB-Paris^K151R^ or 3X Flag vector control into HEK293T cells and then infected with the *ankB* mutants. At 10 hours post-infection, cells were fixed and examined for formation of replicative vacuoles using confocal microscopy. Our data showed that replication of the *ankB* mutant was efficiently *trans*-rescued by ectopically-expressed AnkB-Paris compared to the vector control (Fig. 8a and b). In contrast, replication of the *ankB* mutant was not rescued in cells ectopically expressing AnkB-Paris^K149R^, AnkB-Paris^K151R^, or AnkB-Paris^K149,151R^ substitution mutants. Ectopically expressed AnkB-Paris was localized to the perinuclear ER region while the AnkB-Paris^K149,151R^ was distributed throughout the cytoplasm (Fig. 3). These data indicate that the putative di-lysine ER-retention motif is indispensable for function of AnkB-Paris; likely through membrane anchoring to the ER-derived LCV membrane.

## Discussion

Among the more than 300 confirmed and predicted effectors of *L*. *pneumophila*, very few of them are required for intracellular proliferation and AnkB is one the effectors indispensable for intracellular proliferation^56^. These have been an emerging common theme of variations in the number of effectors and their paralogues among various strains and phenotypic differences between various strains associated with these differences^56, 57^. Loss of the AnkB AA100/130b effector results in a more severe intracellular defect in macrophage and amoeba and *in vivo* ^25, 45^ compared to AnkB-Paris^26^, despite the observations that both function similarly in decorating the LCV with polyubiquitinated proteins. Although host proteasomal degradation is essential for intracellular replication of the Philadelphia-derived Lp02 strain^31^, its AnkB homologue does not contribute to decoration of the LCV with polyubiquitinated proteins or intracellular replication^28^. This suggests that other F-box proteins or ubiquitin ligases, such as SidE and LubX, are involved in decorating the LCV with polyubiquitinated proteins^58–61^. It is also becoming clear that *L*. *pneumophila* translocate deubiquitinases that remove ubiquitin from the modified protein, and variation in translocated deubiquitinases between various *L*. *pneumophila* isolates is likely to be a contributing factor for differences between them in polyubiquitination of the vacuoles and the effectors and metaeffectors (effectors of effectors) involved^60^. Whether metaeffectors of AnkB varies between various isolates is not known. In addition, modification of AnkB by K^11^-linked polyubiquitination and by asparagine hydroxylation has been shown for AnkB-AA100 but it is not known how that differs between isolates^32, 46^. Considering the phenotypic differences between isolates as a consequence of the loss of AnkB and the structural differences in AnkB between the two characterized strains Paris and AA100/130b, it is important to decipher the biological bases of these differences for one of the very few effectors required for intracellular proliferation of *L*. *pneumophila*.

Despite the frame shift mutation and deletion of the C-terminal CaaX farnesylation motif, AnkB-Paris (AnkB1) is required for decoration of the LCV with polyubiquitinated proteins^26^. Concurrently, a unique NKYAP sequence ER retention motif is generated at amino acids 150–154 of AnkB-Paris. The crystal structure of AnkB indicates that the third ankyrin repeat is truncated in AnkB-Paris^48^. Each ankyrin repeat domain is composed of two α-helices connected by a β-loop where the substrate binding domain is located^62^. Our phylogenetic data show that the *ankB1* allele is predominant among environmental isolates. Statistical support for positive selection in *ankB* codons and lineages, and variable effects of *ankB* genotype on recruitment of polyubiquitinated proteins suggest that AnkB may be functionally pleiotropic and may engage diverse cellular pathways triggered by various strains to ensure survival during intracellular residency. Other possibilities include differential regulation of *ankB* in different isolates; read through of the stop codon (encoding a modified aa in place of stop codon) resulting in a full-length functional AnkB similar to AnkB-AA100/130b. We conclude that positive selection acts on few *ankB* codons; that the *ankB1* allele itself is maintained in natural populations by positive selection specifically on the NKYAP ER retention motif; and that the relatively high frequency of the *ankB1* allele in environmental isolates likely reflects a functionally advantageous trait conferred by the *ankB1* allele. The selective advantage to harbor the *ankB1* allele among environmental isolates of *L*. *pneumophila* could be due to a more efficient anchoring to the LCV membrane through the di-lysine ER retention motif compared to farnesylation in some unicellular hosts and/or the third ankyrin domain that is truncated in AnkB1 does not interact with host targets in environmental host but interacts with a specific human target. It is also possible that other effectors expressed by various strains may compensate for the loss of the third ankyrin domain in the *ankB-Paris* allele. Identification of the AnkB-interacting targets and their interacting domains in AnkB should facilitate deciphering these possibilities.

Despite the lack of the farnesylation motif, AnkB-Paris is anchored to the cytosolic side of the LCV membrane. However, substitutions of the di-lysine ER retention motif results in failure to translocate the effector. This indicates an overlap in the signal for membrane anchoring and Dot/Icm-mediated translocation of the AnkB-Paris effector. Unfortunately, it is not possible to determine whether the di-lysine ER retention motif of AnkB-Paris was responsible for localization to the ER-derived LCV membrane, since the di-lysine ER-retention motif substitution in AnkB-Paris resulted in loss of translocation by the Dot/Icm system. In addition, the perinuclear ER-like distribution of ectopically-expressed AnkB-Paris is lost upon alternation of the di-lysine ER retention motif. Importantly, the *trans*-rescue of the *ankB* mutant within cells ectopically expressing AnkB-Paris and the failure of the ER retention di-lysine mutant in *trans*-rescue clearly shows that the ER retention di-lysine motif is essential for the function of AnkB-Paris. This may not be surprising, since substitution of the cysteine within the CaaX farnesylation motif AnkB-AA100 results in a total loss of function of the effector in *trans*-rescue of the *ankB* mutant for intra-vacuolar proliferation^44^. We conclude that anchoring of AnkB variants to host membranes is essential for function, regardless of the mechanism of membrane anchoring by farnensylation or by the di-lysine ER-retention motif.

## Materials and Methods

### Bacterial strains, cell cultures, and infections

*L*. *pneumophila* strain AA100/130b (ATCC BAA-74), its isogenic *dotA* and *ankB* mutants, and complemented mutants were grown on BCYE agar plates for 3–4 days at 37 °C prior to infection as previously described. When required, antibiotics were used at a concentration of 50 µg/mL for kanamycin and 5 µg/mL for chloramphenicol. The *E*. *coli* strain DH5α was used for cloning. *E*. *coli* was grown in Luria-Bertani (LB) and antibiotics were used at a concentration of 100 µg/mL for ampicillin and 40 µg/mL for chloramphenicol. HEK293T cell line was maintained in DMEM (Gibco, Grand Island, NY) supplemented with 10% FBS.

Purification and preparation of human monocyte-derived macrophages (hMDMs) was performed as previously described. Monocytes were isolated from whole blood of healthy donors and then allowed to adhere to 6 well low adherence cell culture plates for 3 days at 37 °C and 5% CO_2_ in RPMI 1640 supplemented with 20% FBS. Monocytes were then counted and re-suspended RPMI 1640 supplemented with 10% FBS and plated on coverslips at a density of 2 × 10^5^ cells per well of a 24 well cell culture plate and incubated for a further 2 days. The cell culture media was then replaced with RPMI 1640 supplemented with 5% FBS for one day, and then with RPMI 1640 supplemented with 1% FBS for one day. The resulting hMDMs were then used for infection.

All methods were carried out in accordance with relevant guidelines and regulations. We confirm that all experimental protocols were approved by the Institutional Review Board (IRB) committee. We confirm that informed consent was obtained from all subjects, as required per our approved IRB protocol.

Infection of hMDMs was performed as previously described. Bacteria were resuspended in RPMI 1640 with 10% FBS and macrophages were infected in triplicate for 1 hour at a multiplicity of infection (MOI) of 10. Plates were centrifuged at 200 g for 5 minutes to synchronize the infection. Infected cells were treated with 50 µg/mL gentamicin for 1 hour to kill extracellular bacteria. Following gentamicin treatment, cells were washed three times with Hank’s buffered saline solution (HBSS) and then RPMI containing 10% FBS was added. At 10 hours post infection, cells were fixed and processed for confocal microscopy. Infection of HEK-293 cells was performed at an MOI of 50 for 1 hour followed by treatment with gentamicin 50 µg/mL for 1 hour. At 10 hours post infection, cells were fixed and processed for confocal microscopy.

### HEK293T cell transfection and infection

To create *ankB*-Paris and *ankB*-ParisK^149,151^R, *ankB* from strain AA100/130b cloned into the mammalian vector p3XFlag-CMV-10 (Sigma) was used as a template for site directed mutagenesis by PCR. HEK293T cells (85% confluent) were re-plated onto poly-L lysine treated coverslips in 24 well plates at a density of 5 × 10^4^ cells/well. After overnight incubation, cells were transfected with 0.625 µg plasmid DNA per well using 1.5 µg polyethylenimine (PEI) per well. After 24 hours, cells were infected with bacteria suspended in DMEM at an MOI of 100 for 1 hour at 37 °C and 5% CO_2_. Plates were centrifuged at 200 g for 10 minutes to synchronize the infection. Extracellular bacteria were eliminated by treatment with gentamicin 50 µg/mL for 1 hour. At 10 hours post infection, cells were fixed and processed for confocal microscopy.

### LCV AnkB localization

To the determine localization of AnkB on the LCV surface during infection, post-nuclear supernatants of infected hMDMs were prepared and then differentially labeled as described previously^44^. Briefly, a total of 1 × 10^6^ hMDMs were infected with *L*. *pneumophila* at an MOI of 10 for 2 h. Post nuclear supernatants were prepared as described previously^44^, and LCVs were allowed to adhere to poly-L-lysine coated glass coverslips and fixed using 4% paraformaldehyde. To differentiate between intact LCVs and extracellular bacteria, the LCVs were labeled prior to permeabilization with mouse anti-*Legionella* antisera (1/1000 dilution) and rabbit anti-AnkB antisera (1/200 dilution) for 1 h. LCVs were then permeabilized with −20 °C methanol and counter-labeled with goat anti-*Legionella* antisera (1/1000 dilution) for 1 h to detect intact LCVs. The LCVs were then labeled with Alexa-Fluor conjugated secondary antibodies (anti-mouse 488, anti-rabbit 555 and anti-goat 647) following the manufacturers recommendations (Invitrogen).

### Confocal microscopy

Processing of infected cells for confocal microscopy was performed as we described previously^44^. Purification of the LCVs and their labeling prior to permeabilization to localize AnkB on the cytosolic side of the LCV membrane was performed as we described previously^44^. For antibody labeling, goat polyclonal anti-*L*. *pneumophila* was used at a dilution of 1:500 and detected by Alexa-Fluor 488-conjugated donkey anti-goat IgG (Invitrogen, Carlsbad, CA). Poly-ubiquitinated proteins were detected using mouse anti-polyubiquitin FK1 antibody at a dilution of 1:50 (BIOMOL International/Affiniti, Exeter, United Kingdom), followed by Alexa-Fluor 647-conjugated goat anti-mouse IgM (Invitrogen, Carlsbad, CA). For detection of 3X-Flag tagged proteins during transfection experiments, mouse monoclonal anti-Flag (Sigma) antibodies were used followed by detection with Alexa-Fluor 488-conjugated donkey anti-mouse (Invitrogen, Carlsbad, CA). An Olympus FV1000 laser scanning confocal microscope was used to examine cells as we described previously. On average, 8–15 0.2 µm serial Z sections of each image were captured and stored for further analyses, using Adobe Photoshop CS3.

### Adenylate cyclase and Western blot analysis

*L*. *pneumophila* strain AA100/130b (ATCC BAA-74) or its isogenic *dotA* mutant harboring pCya-AnkB-Paris, pCya empty vector, or pCya-AnkB-Paris with K^149^R or K^151^R were grown on BCYE agar plates for 3–4 days at 37 °C prior to infection as previously described^45^. U937 macrophages differentiated with PMA were infected at MOI 50 in triplicate and plates were centrifuged to synchronize the infection. After 2 hours at 37 °C and 5% CO_2_, cells were washed three times with PBS and lysed by adding 250 µl of 0.1 N HCl containing 0.5% Triton X-100 and incubating at room temperature for 20 minutes. Lysates were assayed for cAMP using the Direct Cyclic AMP Enzyme Immunoassay kit (Enzo Life Sciences, Inc.). Aliquots of bacteria used for infection (1 × 10^8^ bacteria) were lysed by adding SDS-PAGE loading buffer and boiling for 5 minutes. Fusion protein expression was assessed by Western blot using anti-M45 (1:50 dilution) according to standard procedures. Blots were re-probed with anti-CAT (1:2000).

### PCR and Sequencing of *ankB* alleles

The *ankB* gene was amplified with the following primers: ankB1F: 5′-GGATCCCAAGAGATTTTTAG-3′ and ankB1R: 5′-CATTTAACAAACAAGGCACT-3′ using standard PCR conditions. PCR primers were located in genes flanking *ankB*. Briefly, 25 ng of genomic DNA was used as template in a 25 µL PCR reaction containing 1U of Taq polymerase (Midsci, St. Louis MO), 150 µM dNTPs, 20 pm/ml of each primer with the following cycling parameters: 94 °C-5′ – 1 cycle followed by 30 cycles of 94 °C-1′, 55 °C-1, 72 °C 1′ and a final 5 min extension at 72 °C. DNA sequencing was performed on both strands at the University of Washington Sequencing Core, and the sequence data was assembled and edited using the DNASTAR suite (DNASTAR Inc., Madison, WI).

### Phylogenetic Analysis

Maximum likelihood (ML) tree was constructed using MEGA version 6^63^ assuming the TN93 + G substitution model. The percentage of trees in which the associated taxa clustered together was determined by a bootstrap analysis of 1000 trees. Initial tree for the heuristic search was obtained automatically by applying Neighbor-Joining and BioNJ algorithms to a matrix of pairwise distances estimated using the Maximum Composite Likelihood (MCL) approach, and then selecting the topology with superior log likelihood value. A discrete gamma distribution was used to model evolutionary rate differences among sites (5 categories (+*G*, parameter = 0.2388)). Nucleotide sequence data has been submitted to GenBank® and assigned the following accession numbers: KM276667-KM276681.

### Analysis of Selection Pressures

- Site Models: To identify the different selective forces, i.e., negative, neutral or positive selection, that acted upon *ankB* codons during its evolutionary history we tested the fit of the sequence data to several codon-based models implemented in CODEML package of PAML ver 4.7^64^ accessed via its GUI interface PAMLX1.2^65^ essentially as described before^66^. In brief, we used *site models* to determine selective pressures on each *ankB* codon by comparing the differences in the likelihood score of each model’s fit to the sequence data via a series of likelihood ratio tests [LRTs)^64^. To verify or supplement CODEML outcomes, we conducted several other alternate tests including GARD (genetic algorithms for recombination detection) to detect recombination among *ankB* sequences, and SLAC (single-likelihood ancestor counting), FEL (fixed effects likelihood), IFEL (internal fixed effects likelihood), REL (random effects likelihood), and MEME (mixed effects models of evolution), which can each detect positive and negatively selected codon in protein coding genes and can explicitly account and correct for recombination within sequences. All these methods were accessed and their outcomes analyzed via the www.datamonkey.org server^67^.
- Branch Site Models. To determine whether the *ankB1* allele branch experienced positive selection in its evolutionary history we used two versions of the branch-site models A (M2N2) implemented in CODEML (Table S1): (1) M2N2A1, which specifically tested for evidence of positive selection in the clade leading up to *ankB1* and *ankB8; and* (2) M2N2A2, which specifically sought evidence for positive selection in the *ankB1* branch itself. The fit of each model to the data was tested via LRTs with 1 degree of freedom and that measured the difference in the likelihood score of each model (e.g., M2N2A1) with a constrained version whereby ω for the branch suspected to be under positive selection was fixed at 1 (e.g. M2N2A1ωf). It has been suggested that selection of branches of interest to test for selection, or testing one branch at a time can sometimes lead to statistical instability or acceptance of poorly supported models^67^. Thus to confirm the outcomes of our CODEML branch site results, we performed supplemental analysis for detecting all branches that may have significantly experienced positive selection in their evolutionary history with the GA (genetic algorithm) branch method implemented at www.datamonkey.org.

## Electronic supplementary material

Supplementary Information
