# Supplementary material for: Divergent evolution of Di-lysine ER retention vs. farnesylation motif-mediated anchoring of the AnkB virulence effector to the Legionella-containing vacuolar membrane
Source: Sci Rep. 2017 Jul 11;7:5123. doi: 10.1038/s41598-017-05211-5 (PMC5506055; doi:10.1038/s41598-017-05211-5)
Supplement: Supplementary file 1 — Supplementary Information [file 41598_2017_5211_MOESM1_ESM.pdf]

Supplementary Information

Divergent evolution of Di-lysine ER retention vs. farnesylation motif-mediated anchoring of the AnkB virulence effector to the *Legionella*-containing vacuolar membrane

[1]John D Perpich, [2]Awdhesh Kalia*, [1]Christopher T D Price, [1]Snake C Jones, [3]Kathy Wong, [3]Kalle Gehring, and [1,4]Yousef Abu Kwaik*

Inventory of Tables.

1. **Table S1. Episodic Positive Selection is Observed, Although Purifying or Negative Selection Dominates ankB Evolution: ML Parameter Estimates from CODEML**
2. **Table S2. Detection of recombination usinf GARD analysis**
3. **Table S3: Identification of Codon Sites Under Positive or Negative Selection: FEL Estimates**
4. **Table S4: Identification of Codon Sites Under Positive or Negative Selection: IFEL Estimates**
5. **Table S5: Identification of Codon Sites Under Negative Selection: REL Estimates**
6. **Table S6: Identification of Codon Sites Under Episodic Positive Selection: MEME Estimates**
7. **Table S7. GA Branch analysis for predicting lineages subject to positive selection in a phylogeny.**

Table S1. Purifing or Negative Selection Constrains AnkB evolution: Maximum-likelihood Parameter Estimates from CODONML

Model Code	-*lnL* *	Tree-length	κ[Ŧ]	d_N/d_S	Parameter Esitmates	D [d.f.][¶]	$\chi 2$	P-value	Comments
**M0 (1-ratio)**	1169.58	0.671	2.12	0.163	ω=0.163	NA	NA	NA	NA
**M1a (Nearly Neutral)**	1159.77	0.711	2.04	0.176	p0=0.876, ω0=0.123 p1=0.060, ω1=1	NA	NA	NA	NA
**M2a (Positive Selection)**	1158.34	0.752	2.11	0.228	p0=0.903, ω0=0.077 p1=0.081, ω1=1; [p_S]=0.015, [ω_S]=5.02	M0 *vs* M2a [3] M1a *vs* M2a [2]	19.8 2.6	<0.00018 **0.46 (NS)**	**72L, 86A, 153A** [Pr{ω>}>50<90]; **However, M2a is rejected in favor of M1a**
**M3 (Discrete)**	1158.104	0.758	2.11	0.228	p0=0.593, ω0=0.000; p1=0.387, ω1=0.348; [p_S]=0.019, [ω_S]=4.75	M0 *vs* M3 [4] M1a *vs* M3 [4]	22.52 3.4	<0.00016 **0.49 (NS)**	**72L, 86A, 153A** [Pr{>50<90}]; **However, M3 is rejected in favor of M1a**
**M7 (β)**	1160.33	0.71	2.08	0.183	p=0.117, q=0.525	NA	NA	NA	NA
**M8 (β, ω_S>1)**	1158.144	0.757	2.19	0.225	p=0.201, q=1.797 p0=0.981, [p_S]=0.018, [ω_S]=4.78	M7 *vs* M8 [2]	4.38	**0.112 (MS)**	15V, **72L, 86A, 153A** [Pr{>50<90}]; However, **M8 is rejected in favor of M7**

* ***lnL*, Log Likelihood Score**

Ŧ, κ, kappa, ratio of transition to transversions

¶, D, hierarchial Likelihood Ratio Test statistic and d.f., degrees of freedom

Table S2: Identification of Codon Sites Under Negative Selection: SLAC Estimates

Codon	Observed S Changes	Observed NS Changes	E[S Sites]	E[NS Sites]	Observed S. Prop.	P{S}	dS	dN	dN-dS	P{NS >= observed}	P{NS <= observed}	Normalized dN-dS
1	0	0	0	1.03175	0	0	0	0	0	0	0	0
2	0	0	0.662652	2.25315	0	0.227262	0	0	0	0	0	0
3	1	0	0.780233	2.13557	1	0.267588	1.282	0	-1.282	1	0.267588	-5.80492
4	0	0	0.680765	2.44953	0	0.217476	0	0	0	0	0	0
5	0	0	0.680765	2.44953	0	0.217476	0	0	0	0	0	0
6	0	0	0.680765	2.44953	0	0.217476	0	0	0	0	0	0
7	0	0	1.04343	2.08687	0	0.333333	0	0	0	0	0	0
8	1	1	0.786955	2.34334	0.5	0.2514	1.271	0.427	-0.844	0.936798	0.439597	-3.82254
9	0	0	1.04343	2.08687	0	0.333333	0	0	0	0	0	0
10	0	0	1.04343	2.08687	0	0.333333	0	0	0	0	0	0
11	2	0	0.762558	2.24841	1	0.25326	2.623	0	-2.623	1	**0.0641409**	-11.8789
12	0	0	0.662652	2.34831	0	0.22008	0	0	0	0	0	0
13	0	0	1.04343	2.08687	0	0.333333	0	0	0	0	0	0
14	0	0	0.92949	2.20081	0	0.296934	0	0	0	0	0	0
15	2	2	1.00911	2.12119	0.5	0.322368	1.982	0.943	-1.039	0.898396	0.387918	-4.70621
16	0	0	0.680765	2.44953	0	0.217476	0	0	0	0	0	0
17	0	0	1.04343	2.08687	0	0.333333	0	0	0	0	0	0
18	1	0	1.461	1.4009	1	0.510499	0.684	0	-0.684	1	0.510499	-3.10007
19	0	0	0.680765	2.44953	0	0.217476	0	0	0	0	0	0
20	1	0	0.680765	2.44953	1	0.217476	1.469	0	-1.469	1	0.217476	-6.65309
21	0	0	1.34342	1.42421	0	0.485403	0	0	0	0	0	0
22	0	0	0.662652	2.25315	0	0.227262	0	0	0	0	0	0
23	1	0	1.04343	2.08687	1	0.333333	0.958	0	-0.958	1	0.333333	-4.34066
24	0	1	0.677638	2.41563	0	0.219069	0	0.414	0.414	0.780931	0.219069	1.87495
25	2	0	1.04343	2.08687	1	0.333333	1.917	0	-1.917	1	0.111111	-8.68133
26	2	0	1.71928	1.40633	1	0.550062	1.163	0	-1.163	1	0.302568	-5.2687
27	1	1	1.04343	2.08687	0.5	0.333333	0.958	0.479	-0.479	0.888889	0.555556	-2.17033
28	0	0	1.04343	2.08687	0	0.333333	0	0	0	0	0	0
29	0	1	0.574808	2.55549	0	0.183627	0	0.391	0.3913	0.816373	0.183627	1.77234
30	0	0	1.04343	2.08687	0	0.333333	0	0	0	0	0	0
31	0	0	0.804767	1.64477	0	0.328539	0	0	0	0	0	0

32	0	0	1.04343	2.08687	0	0.333333	0	0	0	0	0	0
33	1	1	0.685342	2.19937	0.5	0.237577	1.459	0.455	-1.004	0.943557	0.418711	-4.54935
34	0	0	0.662652	1.78688	0	0.270522	0	0	0	0	0	0
35	0	0	0.680765	2.44953	0	0.217476	0	0	0	0	0	0
36	0	0	0.680765	2.44953	0	0.217476	0	0	0	0	0	0
37	0	0	0.680765	2.44953	0	0.217476	0	0	0	0	0	0
38	1	0	1.04343	2.08687	1	0.333333	0.958	0	-0.958	1	0.333333	-4.34066
39	1	0	1.49421	1.39641	1	0.516918	0.669	0	-0.669	1	0.516918	-3.03116
40	0	1	1.04343	2.08687	0	0.333333	0	0.479	0.4792	0.666667	0.333333	2.17033
41	0	0	0.680765	2.44953	0	0.217476	0	0	0	0	0	0
42	0	0	0.680765	2.44953	0	0.217476	0	0	0	0	0	0
43	0	0	0.662652	2.25315	0	0.227262	0	0	0	0	0	0
44	0	0	0.680765	2.44953	0	0.217476	0	0	0	0	0	0
45	0	1	1.71561	1.41469	0	0.548065	0	0.707	0.7069	0.451935	0.548065	3.20154
46	1	0	0.663963	2.347	1	0.220515	1.506	0	-1.506	1	0.220515	-6.82145
47	0	0	1.7242	1.4061	0	0.550809	0	0	0	0	0	0
48	0	1	0.680765	2.44953	0	0.217476	0	0.408	0.4082	0.782524	0.217476	1.849
49	0	0	0.662652	1.78688	0	0.270522	0	0	0	0	0	0
50	1	1	1.5052	1.6251	0.5	0.480848	0.664	0.615	-0.049	0.768786	0.730481	-0.222017
51	1	0	0.853475	2.09936	1	0.289036	1.172	0	-1.172	1	0.289036	-5.30676
52	0	0	0.662652	1.78688	0	0.270522	0	0	0	0	0	0
53	2	0	0.775608	1.67392	1	0.316635	2.579	0	-2.579	1	0.100258	-11.6791
54	0	1	1.02103	2.10927	0	0.326177	0	0.474	0.4741	0.673823	0.326177	2.14728
55	0	0	0.380781	2.74952	0	0.121644	0	0	0	0	0	0
56	1	0	0.763155	2.15265	1	0.261731	1.31	0	-1.31	1	0.261731	-5.93482
57	0	1	1.10784	1.3417	0	0.452264	0	0.745	0.7453	0.547736	0.452264	3.37572
58	2	0	0.784203	2.22676	1	0.260449	2.55	0	-2.55	1	**0.0678337**	-11.5511
59	0	0	1.7242	1.4061	0	0.550809	0	0	0	0	0	0
60	0	0	0	1.52076	0	0	0	0	0	0	0	0
61	0	0	1.04343	1.96753	0	0.346544	0	0	0	0	0	0
62	0	0	0.680765	2.44953	0	0.217476	0	0	0	0	0	0
63	0	0	1.04343	2.08687	0	0.333333	0	0	0	0	0	0
64	0	0	0	3.1303	0	0	0	0	0	0	0	0
65	1	2	0.905273	2.22502	0.333333	0.289197	1.105	0.899	-0.206	0.797469	0.640873	-0.931986

66	2	0	1.04343	2.08687	1	0.333333	1.917	0	-1.917	1	0.111111	-8.68133
67	0	0	1.04343	2.08687	0	0.333333	0	0	0	0	0	0
68	0	0	0.828941	2.08687	0	0.284292	0	0	0	0	0	0
69	0	0	0.680765	2.44953	0	0.217476	0	0	0	0	0	0
70	0	0	0.680765	2.44953	0	0.217476	0	0	0	0	0	0
71	0	0	0.680765	2.44953	0	0.217476	0	0	0	0	0	0
72	0	3	1.45245	1.67785	0	0.463996	0	1.788	1.788	0.153994	0.846006	8.0982
73	0	0	0.662652	2.34831	0	0.22008	0	0	0	0	0	0
74	0	0	0.804767	2.2062	0	0.267279	0	0	0	0	0	0
75	0	0	1.04343	2.08687	0	0.333333	0	0	0	0	0	0
76	0	0	0.662652	2.25315	0	0.227262	0	0	0	0	0	0
77	0	1	0.803196	2.11533	0	0.275206	0	0.473	0.4727	0.724794	0.275206	2.14113
78	0	0	0.92949	2.20081	0	0.296934	0	0	0	0	0	0
79	1	0	1.71356	1.40661	1	0.549189	0.584	0	-0.584	1	0.549189	-2.64315
80	0	0	0.662652	2.25315	0	0.227262	0	0	0	0	0	0
81	1	0	0.687186	2.22862	1	0.235676	1.455	0	-1.455	1	0.235676	-6.59092
82	0	1	1.04071	1.96904	0	0.34578	0	0.508	0.5079	0.65422	0.34578	2.3002
83	1	0	0.92949	2.20081	1	0.296934	1.076	0	-1.076	1	0.296934	-4.87277
84	0	0	0.680765	2.44953	0	0.217476	0	0	0	0	0	0
85	2	0	1.04343	2.08687	1	0.333333	1.917	0	-1.917	1	0.111111	-8.68133
86	0	4	1.03362	2.09668	0	0.330198	0	1.908	1.9078	0.201273	0.798727	8.6407
87	2	1	0.784626	1.68187	0.666667	0.318114	2.549	0.595	-1.954	0.967808	0.239206	-8.85188
88	0	0	1.04343	2.08687	0	0.333333	0	0	0	0	0	0
89	0	0	0.680765	2.44953	0	0.217476	0	0	0	0	0	0
90	0	1	0.680765	2.44953	0	0.217476	0	0.408	0.4082	0.782524	0.217476	1.849
91	3	0	0.680765	2.08687	1	0.245974	4.407	0	-4.407	1	**0.0148822**	-19.9593
92	0	0	0.680765	2.44953	0	0.217476	0	0	0	0	0	0
93	0	0	1.34342	1.42421	0	0.485403	0	0	0	0	0	0
94	0	0	0.680765	2.44953	0	0.217476	0	0	0	0	0	0
95	0	0	0.828941	2.08687	0	0.284292	0	0	0	0	0	0
96	2	0	1.04343	2.08687	1	0.333333	1.917	0	-1.917	1	0.111111	-8.68133
97	0	0	1.04343	2.08687	0	0.333333	0	0	0	0	0	0
98	0	0	1.34342	1.42421	0	0.485403	0	0	0	0	0	0
99	1	0	1.06819	2.06211	1	0.341243	0.936	0	-0.936	1	0.341243	-4.24005

100	0	0	1.04343	2.08687	0	0.333333	0	0	0	0	0	0
101	1	0	1.04343	2.08687	1	0.333333	0.958	0	-0.958	1	0.333333	-4.34066
102	1	0	0.87168	2.25862	1	0.278466	1.147	0	-1.147	1	0.278466	-5.19593
103	0	0	0.662652	2.34831	0	0.22008	0	0	0	0	0	0
104	1	0	1.04343	1.97388	1	0.345815	0.958	0	-0.958	1	0.345815	-4.34066
105	0	2	0.686219	2.22959	0	0.235345	0	0.897	0.897	0.584698	0.415302	4.06281
106	2	0	1.04343	2.08687	1	0.333333	1.917	0	-1.917	1	0.111111	-8.68133
107	0	0	0.680765	2.08687	0	0.245974	0	0	0	0	0	0
108	0	0	0.662652	1.78688	0	0.270522	0	0	0	0	0	0
109	0	0	1.04343	2.08687	0	0.333333	0	0	0	0	0	0
110	3	0	1.04343	2.08687	1	0.333333	2.875	0	-2.875	1	**0.037037**	-13.022
111	1	0	0.680765	2.44953	1	0.217476	1.469	0	-1.469	1	0.217476	-6.65309
112	0	0	0.680765	2.08687	0	0.245974	0	0	0	0	0	0
113	0	0	1.04343	2.08687	0	0.333333	0	0	0	0	0	0
114	0	0	0	1.52076	0	0	0	0	0	0	0	0
115	0	0	0.828941	2.08687	0	0.284292	0	0	0	0	0	0
116	0	0	0.662652	2.25315	0	0.227262	0	0	0	0	0	0
117	0	0	0.680765	2.08687	0	0.245974	0	0	0	0	0	0
118	0	0	1.04343	2.08687	0	0.333333	0	0	0	0	0	0
119	1	0	0.680765	2.44953	1	0.217476	1.469	0	-1.469	1	0.217476	-6.65309
120	0	1	0.682336	2.44645	0	0.218083	0	0.409	0.4088	0.781917	0.218083	1.85133
121	3	0	1.04343	2.08687	1	0.333333	2.875	0	-2.875	1	**0.037037**	-13.022
122	0	0	0.680765	2.44953	0	0.217476	0	0	0	0	0	0
123	0	0	0.680765	2.44953	0	0.217476	0	0	0	0	0	0
124	0	0	0.662652	2.25315	0	0.227262	0	0	0	0	0	0
125	0	0	0.680765	2.44953	0	0.217476	0	0	0	0	0	0
126	0	0	0.680765	2.44953	0	0.217476	0	0	0	0	0	0
127	0	0	0.680765	2.08687	0	0.245974	0	0	0	0	0	0
128	0	0	1.04343	2.08687	0	0.333333	0	0	0	0	0	0
129	0	0	0.680765	2.44953	0	0.217476	0	0	0	0	0	0
130	0	0	1.04343	1.7242	0	0.377013	0	0	0	0	0	0
131	0	0	1.04343	2.08687	0	0.333333	0	0	0	0	0	0
132	0	0	0.92949	2.20081	0	0.296934	0	0	0	0	0	0
133	0	0	1.04343	2.08687	0	0.333333	0	0	0	0	0	0

134	0	0	1.04343	2.08687	0	0.333333	0	0	0	0	0	0
135	0	0	1.04343	2.08687	0	0.333333	0	0	0	0	0	0
136	0	0	0.662652	2.25315	0	0.227262	0	0	0	0	0	0
137	1	0	0.799599	2.11621	1	0.274229	1.251	0	-1.251	1	0.274229	-5.66433
138	0	0	0.662652	1.78688	0	0.270522	0	0	0	0	0	0
139	1	0	1.7242	1.4061	1	0.550809	0.58	0	-0.58	1	0.550809	-2.62684
140	0	0	1.04343	2.08687	0	0.333333	0	0	0	0	0	0
141	0	0	0.680765	2.44953	0	0.217476	0	0	0	0	0	0
142	0	0	1.04343	2.08687	0	0.333333	0	0	0	0	0	0
143	0	0	1.04343	2.08687	0	0.333333	0	0	0	0	0	0
144	0	0	0.680765	2.44953	0	0.217476	0	0	0	0	0	0
145	0	1	0.806785	2.11083	0	0.276522	0	0.474	0.4737	0.723478	0.276522	2.14569
146	0	0	0.680765	2.44953	0	0.217476	0	0	0	0	0	0
147	0	0	0.804767	2.11104	0	0.276002	0	0	0	0	0	0
148	1	0	0.667821	2.34314	1	0.221796	1.497	0	-1.497	1	0.221796	-6.78205
149	0	0	0.662652	2.25315	0	0.227262	0	0	0	0	0	0
150	0	1	0.663528	2.26266	0	0.226755	0	0.442	0.442	0.773245	0.226755	2.00171
151	0	1	0.662652	1.80944	0	0.268053	0	0.553	0.5527	0.731947	0.268053	2.50308
152	0.5	2.5	0.409463	2.70857	0.166667	0.131321	1.221	0.923	-0.298	0.804151	0.344492	-1.35021
153	1	2	1.04544	2.08486	0.333333	0.333974	0.957	0.959	0.0028	0.739886	0.704557	0.0125055
154	1	1	1.04343	2.08687	0.5	0.333333	0.958	0.479	-0.479	0.888889	0.555556	-2.17033
155	1	0	1.22771	1.28354	1	0.488884	0.815	0	-0.815	1	0.488884	-3.68914
156	2	0	0.839532	1.98781	1	0.296934	2.382	0	-2.382	1	**0.0881695**	-10.7898
157	0	1	0.871027	1.94851	0	0.308925	0	0.513	0.5132	0.691075	0.308925	2.32444
158	0	1	0.723398	1.99616	0	0.265999	0	0.501	0.501	0.734001	0.265999	2.26895
159	0	0	0	2.82734	0	0	0	0	0	0	0	0
160	0	0	0.598519	1.61394	0	0.270522	0	0	0	0	0	0
161	1	0	0.600353	2.1192	1	0.220754	1.666	0	-1.666	1	0.220754	-7.54421
162	0	0	0.598519	2.12104	0	0.22008	0	0	0	0	0	0
163	0	0	0.598519	2.03509	0	0.227262	0	0	0	0	0	0
164	0	1	0.368028	2.45931	0	0.130168	0	0.407	0.4066	0.869832	0.130168	1.84165
165	0	1	0.942447	1.88489	0	0.333333	0	0.531	0.5305	0.666667	0.333333	2.40289
166	0	0	0.72688	1.48558	0	0.328539	0	0	0	0	0	0
167	0	1	0.614879	2.21246	0	0.217476	0	0.452	0.452	0.782524	0.217476	2.04713

168	0	1	0.732928	1.90848	0	0.277476	0	0.524	0.524	0.722524	0.277476	2.37319
169	0	0	0.614879	1.98781	0	0.236248	0	0	0	0	0	0
170	0	0	0.942447	1.88489	0	0.333333	0	0	0	0	0	0
171	1	0	0.942447	1.88489	1	0.333333	1.061	0	-1.061	1	0.333333	-4.80578
172	1	0	0.614879	1.98781	1	0.236248	1.626	0	-1.626	1	0.236248	-7.36599

Negatively selected sites are highlighted in yellow and their corresponding significance values are highlighted in red.

Table S3: Identification of Codon Sites Under Positive or Negative Selection: FEL Estimates

Codon	dS	dN	dN/dS	Normalized dN-dS	dS (when dN=dS)	Log(L)	LRT	p-value
1	0	0	Undefined	0	0	0	0	1
2	0	0	Undefined	0	0	0	0	1
3	2.47411	0	0	-11.2051	0.726608	-6.31909	2.3815	0.122779
4	0	0	Undefined	0	0	0	0	1
5	0	0	Undefined	0	0	0	0	1
6	0	0	Undefined	0	0	0	0	1
7	0	0	Undefined	0	0	0	0	1
8	3.39881	0.830619	0.244	-11.6312	1.33026	-14.0149	0.847318	0.357312
9	0	0	Undefined	0	0	0	0	1
10	0	0	Undefined	0	0	0	0	1
**11**	**7.73595**	**0**	**0**	**-35.0358**	**1.48911**	**-10.2682**	**6.97176**	**0.008281**
12	0	0	Undefined	0	0	0	0	1
13	0	0	Undefined	0	0	0	0	1
14	0	0	Undefined	0	0	0	0	1
15	4.19434	2.28716	0.545	-8.63751	2.9371	-18.6777	0.333538	0.563583
16	0	0	Undefined	0	0	0	0	1
17	0	0	Undefined	0	0	0	0	1
18	1.31479	0	0	-5.95463	0.681072	-8.24265	1.29106	0.255852
19	0	0	Undefined	0	0	0	0	1
**20**	**2.87278**	**0**	**0**	**-13.0107**	**0.629082**	**-7.65032**	**2.9457**	**0.086107**
21	0	0	Undefined	0	0	0	0	1
22	0	0	Undefined	0	0	0	0	1
**23**	**2.53342**	**0**	**0**	**-11.4737**	**0.585093**	**-7.09675**	**2.88836**	**0.089222**
24	0	1.01958	Infinite	4.61762	0.741294	-8.64097	0.625994	0.428829
**25**	**6.65713**	**0**	**0**	**-30.1499**	**1.40227**	**-12.5823**	**5.62623**	**0.017694**
**26**	**3.07813**	**0**	**0**	**-13.9407**	**1.46367**	**-12.6083**	**2.79903**	**0.094321**
27	2.43453	0.682511	0.28	-7.93479	1.07125	-16.0971	0.74357	0.38852
28	0	0	Undefined	0	0	0	0	1
29	1.00E-06	0.96427	964270	4.36713	0.748808	-7.83779	0.512731	0.473959
30	0	0	Undefined	0	0	0	0	1
31	0	0	Undefined	0	0	0	0	1
32	0	0	Undefined	0	0	0	0	1
33	7.12973	0.719994	0.101	-29.0294	1.26083	-15.5275	1.82838	0.176319

34	0	0	Undefined	0	0	0	0	1
35	0	0	Undefined	0	0	0	0	1
36	0	0	Undefined	0	0	0	0	1
37	0	0	Undefined	0	0	0	0	1
38	1.84557	0	0	-8.35851	0.558094	-8.33803	2.35362	0.124992
39	1.13509	0	0	-5.14078	0.628254	-8.38707	1.16988	0.279426
40	0	0.830526	Infinite	3.76141	0.61284	-9.79086	0.601829	0.437881
41	0	0	Undefined	0	0	0	0	1
42	0	0	Undefined	0	0	0	0	1
43	0	0	Undefined	0	0	0	0	1
44	0	0	Undefined	0	0	0	0	1
45	1.00E-06	1.47919	1479190	6.6992	0.60941	-10.1237	1.75365	0.185419
**46**	**7.45995**	**0**	**0**	**-33.7858**	**0.716762**	**-8.01311**	**4.01308**	**0.045149**
47	0	0	Undefined	0	0	0	0	1
48	0	1.07132	Infinite	4.85198	0.85287	-8.37389	0.449743	0.502457
49	0	0	Undefined	0	0	0	0	1
50	1.85393	1.30096	0.702	-2.50438	1.52504	-14.2881	0.058044	0.809615
**51**	**3.0806**	**0**	**0**	**-13.9519**	**0.614237**	**-7.14593**	**3.21492**	**0.07297**
52	0	0	Undefined	0	0	0	0	1
53	4.87907	0	0	-22.0971	1.67329	-12.0104	3.9293	0.047452
54	0	0.83154	Infinite	3.76601	0.617782	-9.8516	0.591721	0.441754
55	0	0	Undefined	0	0	0	0	1
**56**	**3.96922**	**0**	**0**	**-17.9764**	**0.8879**	**-6.21242**	**3.28016**	**0.070122**
57	0	1.65612	Infinite	7.50048	1.04116	-7.83727	0.962304	0.326607
**58**	**5.58717**	**0**	**0**	**-25.3041**	**1.27572**	**-9.64661**	**5.53465**	**0.018644**
59	0	0	Undefined	0	0	0	0	1
60	0	0	Undefined	0	0	0	0	1
61	0	0	Undefined	0	0	0	0	1
62	0	0	Undefined	0	0	0	0	1
63	0	0	Undefined	0	0	0	0	1
64	0	0	Undefined	0	0	0	0	1
65	3.11991	1.74449	0.559	-6.22922	2.08971	-18.3287	0.218946	0.639844
**66**	**3.68974**	**0**	**0**	**-16.7107**	**1.07154**	**-11.161**	**4.76635**	**0.029021**
67	0	0	Undefined	0	0	0	0	1
68	0	0	Undefined	0	0	0	0	1

69	0	0	Undefined	0	0	0	0	1
70	0	0	Undefined	0	0	0	0	1
71	0	0	Undefined	0	0	0	0	1
**72**	**0**	**3.91627**	**Infinite**	**17.7366**	**2.01093**	**-18.5672**	**3.79087**	**0.051533**
73	0	0	Undefined	0	0	0	0	1
74	0	0	Undefined	0	0	0	0	1
75	0	0	Undefined	0	0	0	0	1
76	0	0	Undefined	0	0	0	0	1
77	0	1.07169	Infinite	4.85363	0.673033	-10.4273	0.907693	0.340727
78	0	0	Undefined	0	0	0	0	1
79	1.68755	0	0	-7.64286	0.770168	-8.54267	1.5169	0.21809
80	0	0	Undefined	0	0	0	0	1
**81**	**4.11106**	**0**	**0**	**-18.6188**	**0.842038**	**-5.78135**	**3.16119**	**0.075408**
82	0	0.704001	Infinite	3.18839	0.547353	-9.92572	0.500811	0.479144
83	2.06607	0	0	-9.35715	0.664832	-7.07466	2.22949	0.135398
84	0	0	Undefined	0	0	0	0	1
**85**	**6.01955**	**0**	**0**	**-27.2623**	**1.40098**	**-13.1604**	**5.35359**	**0.02068**
**86**	**4.53E-15**	**3.96962**	**8.77E+14**	**17.9782**	**2.40087**	**-17.2705**	**3.82911**	**0.05037**
87	5.08254	1.41404	0.278	-16.6145	2.71966	-12.2546	1.06805	0.301387
88	0	0	Undefined	0	0	0	0	1
89	0	0	Undefined	0	0	0	0	1
90	0	0.730128	Infinite	3.30672	0.539879	-8.95031	0.597208	0.439645
**91**	**14.8419**	**0**	**0**	**-67.2181**	**2.63655**	**-11.9447**	**9.34933**	**0.002231**
92	0	0	Undefined	0	0	0	0	1
93	0	0	Undefined	0	0	0	0	1
94	0	0	Undefined	0	0	0	0	1
95	0	0	Undefined	0	0	0	0	1
**96**	**5.40423**	**0**	**0**	**-24.4755**	**1.37904**	**-10.3291**	**5.2521**	**0.02192**
97	0	0	Undefined	0	0	0	0	1
98	0	0	Undefined	0	0	0	0	1
99	2.76583	0	0	-12.5263	0.785666	-7.45631	2.44825	0.117656
100	0	0	Undefined	0	0	0	0	1
**101**	**2.79821**	**0**	**0**	**-12.673**	**0.592237**	**-8.74395**	**3.00106**	**0.08321**
102	1.98111	0	0	-8.97234	0.650727	-8.89162	2.1941	0.138541
103	0	0	Undefined	0	0	0	0	1

104	2.6862	0	0	-12.1657	0.543026	-10.6205	3.0825	0.079139
105	0	2.09693	Infinite	9.49692	1.65244	-10.277	0.936297	0.333232
106	8.1606	0	0	-36.959	1.26272	-10.4404	6.9204	0.008522
107	0	0	Undefined	0	0	0	0	1
108	0	0	Undefined	0	0	0	0	1
109	0	0	Undefined	0	0	0	0	1
110	12.9434	0	0	-58.6201	1.91863	-12.8224	10.4245	0.001244
111	5.92581	0	0	-26.8377	0.800609	-8.55586	3.60823	0.057494
112	0	0	Undefined	0	0	0	0	1
113	0	0	Undefined	0	0	0	0	1
114	0	0	Undefined	0	0	0	0	1
115	0	0	Undefined	0	0	0	0	1
116	0	0	Undefined	0	0	0	0	1
117	0	0	Undefined	0	0	0	0	1
118	0	0	Undefined	0	0	0	0	1
119	2.41085	0	0	-10.9186	0.608068	-7.3347	2.70802	0.099845
120	4.64E-15	0.805385	1.74E+14	3.64755	0.678311	-11.1954	0.340684	0.559435
121	8.77879	0	0	-39.7587	2.06269	-17.1372	7.63292	0.005731
122	0	0	Undefined	0	0	0	0	1
123	0	0	Undefined	0	0	0	0	1
124	0	0	Undefined	0	0	0	0	1
125	0	0	Undefined	0	0	0	0	1
126	0	0	Undefined	0	0	0	0	1
127	0	0	Undefined	0	0	0	0	1
128	0	0	Undefined	0	0	0	0	1
129	0	0	Undefined	0	0	0	0	1
130	0	0	Undefined	0	0	0	0	1
131	0	0	Undefined	0	0	0	0	1
132	0	0	Undefined	0	0	0	0	1
133	0	0	Undefined	0	0	0	0	1
134	0	0	Undefined	0	0	0	0	1
135	0	0	Undefined	0	0	0	0	1
136	0	0	Undefined	0	0	0	0	1
137	2.06856	0	0	-9.36844	0.692352	-6.00787	2.16103	0.14155
138	0	0	Undefined	0	0	0	0	1

139	1.12686	0	0	-5.10351	0.631058	-7.54913	1.1455	0.284493
140	0	0	Undefined	0	0	0	0	1
141	0	0	Undefined	0	0	0	0	1
142	0	0	Undefined	0	0	0	0	1
143	0	0	Undefined	0	0	0	0	1
144	0	0	Undefined	0	0	0	0	1
145	1.00E-06	1.08049	1080490	4.89351	0.668865	-11.3574	0.935387	0.333467
146	0	0	Undefined	0	0	0	0	1
147	0	0	Undefined	0	0	0	0	1
**148**	**4.50295**	**0**	**0**	**-20.3936**	**0.705398**	**-5.76889**	**3.58461**	**0.058317**
149	0	0	Undefined	0	0	0	0	1
150	0	1.07003	Infinite	4.8461	0.869531	-8.94221	0.408088	0.522941
151	0	1.38533	Infinite	6.27409	1.04524	-8.31136	0.561307	0.453734
152	4.47452	2.31922	0.518	-9.76123	2.61875	-18.477	0.08283	0.773499
153	1.62972	1.8318	1.124	0.915221	1.75945	-21.5782	0.007985	0.928795
154	1.58193	0.809683	0.512	-3.49745	1.07261	-14.5533	0.213783	0.643819
155	2.02965	0	0	-9.19219	0.903146	-9.57851	1.55776	0.211994
**156**	**10.1035**	**0**	**0**	**-45.7585**	**2.34204**	**-10.403**	**7.50478**	**0.006154**
157	0	0.880676	Infinite	3.98854	0.586915	-7.82003	0.801978	0.370503
158	0	1.02908	Infinite	4.66067	0.819459	-10.1309	0.439684	0.507275
159	0.921916	0	0	-4.17532	3.18E-16	-4.08746	0	1
160	0	0	Undefined	0	9.56E-17	-3.32328	0	1
**161**	**10.1426**	**0**	**0**	**-45.9354**	**0.79446**	**-7.56531**	**4.0349**	**0.044568**
162	0	0	Undefined	0	9.56E-17	-3.30648	0	1
163	0	0	Undefined	0	3.18E-16	-2.81415	0	1
164	0	0.990699	Infinite	4.48683	0.838291	-7.02306	0.325756	0.568169
165	0	0.90089	Infinite	4.08009	0.677649	-7.24055	0.566293	0.451736
166	0	0	Undefined	0	9.68E-17	-4.15973	0	1
167	0	0.817747	Infinite	3.70354	0.603604	-7.89788	0.600162	0.438516
168	0	1.16583	Infinite	5.27997	0.727603	-7.5452	0.926557	0.335759
169	0	0	Undefined	0	9.56E-17	-5.62162	0	1
170	0	0	Undefined	0	3.18E-16	-3.91262	0	1
**171**	**3.46287**	**0**	**0**	**-15.6832**	**0.732629**	**-7.22602**	**3.01643**	**0.082425**
**172**	**5.13403**	**0**	**0**	**-23.2518**	**0.680606**	**-7.26866**	**3.90818**	**0.048052**

Negatively selected sites are highlighted in yellow and their corresponding significance values are highlighted in red.

Positively selected sites are highlighted in purple and their corresponding signficance value are shown in orange text

Table S4: Identification of Codon Sites Under Positive or Negative Selection: IFEL Estimates

Codon (N-1)	dS	dN	dN Leaves	dN/dS	Normalized dN-dS	dS (when dN=dS)	Log(L)	LRT	p-value
2	0	0	0	Undefined	0	0	0	0	1
3	0	0	0	Undefined	0	0	0	0	1
4	2.47404	0	0	0	-11.2048	0.968522	-6.31909	1.82465	0.176761
5	0	0	0	Undefined	0	0	0	0	1
6	0	0	0	Undefined	0	0	0	0	1
7	0	0	0	Undefined	0	0	0	0	1
8	0	0	0	Undefined	0	0	0	0	1
9	3.40809	1.34757	0	0.395	-9.332	1.94162	-13.5547	0.370422	0.542775
10	0	0	0	Undefined	0	0	0	0	1
11	0	0	0	Undefined	0	0	0	0	1
**12**	**7.73595**	**0**	**0**	**0**	**-35.0358**	**2.15147**	**-10.2682**	**5.52211**	**0.018778**
13	0	0	0	Undefined	0	0	0	0	1
14	0	0	0	Undefined	0	0	0	0	1
15	0	0	0	Undefined	0	0	0	0	1
16	4.19738	2.01717	2.656	0.481	-9.87411	3.05321	-18.6598	0.354244	0.551721
17	0	0	0	Undefined	0	0	0	0	1
18	0	0	0	Undefined	0	0	0	0	1
19	1.31479	0	0	0	-5.95463	0.818253	-8.24265	0.931082	0.334582
20	0	0	0	Undefined	0	0	0	0	1
21	2.87269	0	0	0	-13.0103	0.862031	-7.65032	2.3266	0.12718
22	0	0	0	Undefined	0	0	0	0	1
23	0	0	0	Undefined	0	0	0	0	1
24	2.53349	0	0	0	-11.4741	0.799884	-7.09675	2.27321	0.131627
25	0	1.63589	0	Infinite	7.40888	1.01491	-8.19481	0.91257	0.339433
**26**	**6.65713**	**0**	**0**	**0**	**-30.1499**	**1.94143**	**-12.5823**	**4.40968**	**0.035736**
27	3.07803	0	0	0	-13.9402	1.78928	-12.6083	2.0365	0.153563
28	2.43447	0	2.02771	0	-11.0256	0.723895	-15.0118	2.41599	0.120102
29	0	0	0	Undefined	0	0	0	0	1
30	0	1.61392	0	Infinite	7.30938	1.09597	-7.33206	0.78334	0.376122
31	0	0	0	Undefined	0	0	0	0	1
32	0	0	0	Undefined	0	0	0	0	1
33	0	0	0	Undefined	0	0	0	0	1
**34**	**7.13035**	**0**	**2.11299**	**0**	**-32.293**	**0.888479**	**-14.4537**	**3.62091**	**0.057058**

35	0	0	0	Undefined	0	0	0	0	1
36	0	0	0	Undefined	0	0	0	0	1
37	0	0	0	Undefined	0	0	0	0	1
38	0	0	0	Undefined	0	0	0	0	1
39	1.84557	0	0	0	-8.35851	0.735796	-8.33803	1.80823	0.178721
40	1.13511	0	0	0	-5.14087	0.744934	-8.38707	0.833804	0.361175
41	2.58E-15	0	2.28953	0	-1.17E-14	0	-8.78026	0	1
42	0	0	0	Undefined	0	0	0	0	1
43	0	0	0	Undefined	0	0	0	0	1
44	0	0	0	Undefined	0	0	0	0	1
45	0	0	0	Undefined	0	0	0	0	1
46	0	0	4.29703	Undefined	0	1.00E-06	-9.06233	2.84E-06	0.998654
**47**	**7.45995**	**0**	**0**	**0**	**-33.7858**	**1.03051**	**-8.01311**	**3.31196**	**0.068777**
48	0	0	0	Undefined	0	0	0	0	1
49	3.66E-15	1.76674	0	4.83E+14	8.00149	1.23643	-7.90095	0.683313	0.408448
50	0	0	0	Undefined	0	0	0	0	1
51	1.85966	2.06726	0	1.112	0.940226	1.95835	-13.8537	0.005054	0.943323
52	3.0806	0	0	0	-13.9519	0.856339	-7.14593	2.5602	0.109585
53	0	0	0	Undefined	0	0	0	0	1
**54**	**4.87907**	**0**	**0**	**0**	**-22.0971**	**2.16452**	**-12.0104**	**2.95333**	**0.085701**
55	0	1.27767	0	Infinite	5.78653	0.833384	-9.43163	0.843461	0.358409
56	0	0	0	Undefined	0	0	0	0	1
57	3.96922	0	0	0	-17.9764	1.25694	-6.21242	2.58131	0.108132
58	0	2.9042	0	Infinite	13.153	1.45528	-7.22291	1.49211	0.221889
**59**	**5.58736**	**0**	**0**	**0**	**-25.3049**	**1.75763**	**-9.64661**	**4.3203**	**0.03766**
60	0	0	0	Undefined	0	0	0	0	1
61	0	0	0	Undefined	0	0	0	0	1
62	0	0	0	Undefined	0	0	0	0	1
63	0	0	0	Undefined	0	0	0	0	1
64	0	0	0	Undefined	0	0	0	0	1
65	0	0	0	Undefined	0	0	0	0	1
66	3.12375	1.36784	2.35459	0.438	-7.95244	1.97996	-18.2598	0.338218	0.56086
**67**	**3.68974**	**0**	**0**	**0**	**-16.7107**	**1.42778**	**-11.161**	**3.6579**	**0.055804**
68	0	0	0	Undefined	0	0	0	0	1
69	0	0	0	Undefined	0	0	0	0	1

70	0	0	0	Undefined	0	0	0	0	1
71	0	0	0	Undefined	0	0	0	0	1
72	0	0	0	Undefined	0	0	0	0	1
73	0	1.17072	9.557	Infinite	5.30213	5.31E-15	-17.6607	0.451072	0.501826
74	0	0	0	Undefined	0	0	0	0	1
75	0	0	0	Undefined	0	0	0	0	1
76	0	0	0	Undefined	0	0	0	0	1
77	0	0	0	Undefined	0	0	0	0	1
78	0	0	3.15989	Undefined	0	0	-9.35388	0	1
79	0	0	0	Undefined	0	0	0	0	1
80	1.68755	0	0	0	-7.64286	0.946837	-8.54267	1.11482	0.291038
81	0	0	0	Undefined	0	0	0	0	1
82	4.11107	0	0	0	-18.6188	1.18367	-5.78135	2.50069	0.113796
83	1.44E-15	0	2.06641	0	-6.50E-15	0	-8.85249	0	1
84	2.06607	0	0	0	-9.35715	0.873282	-7.07466	1.69675	0.192714
85	0	0	0	Undefined	0	0	0	0	1
**86**	**6.01955**	**0**	**0**	**0**	**-27.2623**	**1.90707**	**-13.1604**	**4.17962**	**0.040913**
**87**	**1.23E-15**	**7.24507**	**0**	**5.91E+15**	**32.8126**	**3.22126**	**-15.2617**	**5.70698**	**0.016898**
88	5.08069	2.30403	0	0.453	-12.5753	3.64289	-11.7912	0.381882	0.536598
89	0	0	0	Undefined	0	0	0	0	1
90	0	0	0	Undefined	0	0	0	0	1
91	0	1.10724	0	Infinite	5.01464	0.719632	-8.54477	0.842468	0.358692
**92**	**14.8419**	**0**	**0**	**0**	**-67.2181**	**3.77457**	**-11.9447**	**7.36927**	**0.006635**
93	0	0	0	Undefined	0	0	0	0	1
94	0	0	0	Undefined	0	0	0	0	1
95	0	0	0	Undefined	0	0	0	0	1
96	0	0	0	Undefined	0	0	0	0	1
**97**	**5.4044**	**0**	**0**	**0**	**-24.4763**	**1.88545**	**-10.3291**	**4.06199**	**0.043859**
98	0	0	0	Undefined	0	0	0	0	1
99	0	0	0	Undefined	0	0	0	0	1
100	2.76583	0	0	0	-12.5263	1.05585	-7.45631	1.87122	0.171335
101	0	0	0	Undefined	0	0	0	0	1
102	2.79821	0	0	0	-12.673	0.814488	-8.74395	2.37673	0.123155
103	1.98111	0	0	0	-8.97234	0.859535	-8.89162	1.64928	0.199057
104	0	0	0	Undefined	0	0	0	0	1

105	2.6862	0	0	0	-12.1657	0.749493	-10.6205	2.44996	0.117528
106	2.93E-15	3.56762	0	1.22E+15	16.1576	2.44516	-9.44244	1.41547	0.234151
**107**	**8.1606**	**0**	**0**	**0**	**-36.959**	**1.79842**	**-10.4404**	**5.5756**	**0.018213**
108	0	0	0	Undefined	0	0	0	0	1
109	0	0	0	Undefined	0	0	0	0	1
110	0	0	0	Undefined	0	0	0	0	1
**111**	**12.9434**	**0**	**0**	**0**	**-58.6201**	**2.78242**	**-12.8224**	**8.37738**	**0.003799**
**112**	**5.9256**	**0**	**0**	**0**	**-26.8368**	**1.14161**	**-8.55586**	**2.92757**	**0.087079**
113	0	0	0	Undefined	0	0	0	0	1
114	0	0	0	Undefined	0	0	0	0	1
115	0	0	0	Undefined	0	0	0	0	1
116	0	0	0	Undefined	0	0	0	0	1
117	0	0	0	Undefined	0	0	0	0	1
118	0	0	0	Undefined	0	0	0	0	1
119	0	0	0	Undefined	0	0	0	0	1
120	2.41085	0	0	0	-10.9186	0.822206	-7.3347	2.11321	0.146032
121	2.35E-15	0	2.36935	0	-1.06E-14	0	-10.1215	-1.78E-14	1
**122**	**8.77879**	**0**	**0**	**0**	**-39.7587**	**2.80616**	**-17.1372**	**5.91335**	**0.015027**
123	0	0	0	Undefined	0	0	0	0	1
124	0	0	0	Undefined	0	0	0	0	1
125	0	0	0	Undefined	0	0	0	0	1
126	0	0	0	Undefined	0	0	0	0	1
127	0	0	0	Undefined	0	0	0	0	1
128	0	0	0	Undefined	0	0	0	0	1
129	0	0	0	Undefined	0	0	0	0	1
130	0	0	0	Undefined	0	0	0	0	1
131	0	0	0	Undefined	0	0	0	0	1
132	0	0	0	Undefined	0	0	0	0	1
133	0	0	0	Undefined	0	0	0	0	1
134	0	0	0	Undefined	0	0	0	0	1
135	0	0	0	Undefined	0	0	0	0	1
136	0	0	0	Undefined	0	0	0	0	1
137	0	0	0	Undefined	0	0	0	0	1
138	2.06856	0	0	0	-9.36844	0.906891	-6.00787	1.63447	0.201086
139	0	0	0	Undefined	0	0	0	0	1

140	1.12688	0	0	0	-5.1036	0.744723	-7.54913	0.819052	0.365458
141	0	0	0	Undefined	0	0	0	0	1
142	0	0	0	Undefined	0	0	0	0	1
143	0	0	0	Undefined	0	0	0	0	1
144	0	0	0	Undefined	0	0	0	0	1
145	0	0	0	Undefined	0	0	0	0	1
146	0	0	3.21596	Undefined	0	0	-10.2749	0	1
147	0	0	0	Undefined	0	0	0	0	1
148	0	0	0	Undefined	0	0	0	0	1
**149**	**4.50295**	**0**	**0**	**0**	**-20.3936**	**1.00416**	**-5.76889**	**2.89694**	**0.088748**
150	0	0	0	Undefined	0	0	0	0	1
151	5.43E-15	0	3.13285	0	-2.46E-14	0	-7.87497	-1.78E-15	1
152	0	0	4.13246	Undefined	0	0	-7.22229	1.78E-15	1
153	2.34E-14	0	9.9671	0	-1.06E-13	1.85E-16	-15.1985	1.78E-14	1
154	7.77E-15	0	11.6181	0	-3.52E-14	5.70E-15	-18.6121	2.13E-14	1
155	1.58188	0	2.5724	0	-7.16425	0.67163	-13.3986	1.69846	0.192489
156	2.02965	0	0	0	-9.19219	1.06488	-9.57851	1.23885	0.265693
**157**	**10.1035**	**0**	**0**	**0**	**-45.7585**	**3.09528**	**-10.403**	**6.18795**	**0.012862**
158	0	1.25426	0	Infinite	5.68048	0.72992	-7.47827	1.0575	0.303786
159	0	1.5646	0	Infinite	7.08602	1.11654	-9.74575	0.625863	0.428877
160	0.922851	0	0	0	-4.17955	0	-4.08746	0	1
161	0	0	0	Undefined	0	0	-3.32328	0	1
**162**	**10.1426**	**0**	**0**	**0**	**-45.9354**	**1.04575**	**-7.56531**	**3.5074**	**0.061095**
163	0	0	0	Undefined	0	0	-3.30648	0	1
164	0	0	0	Undefined	0	5.55E-17	-2.81415	0	1
165	1.02E-15	1.42243	0	1.4E+15	6.44214	1.12337	-6.67936	0.449016	0.502802
166	7.62E-16	1.27584	0	1.67E+15	5.77822	0.868905	-6.90259	0.756444	0.384444
167	0	0	0	Undefined	0	0	-4.15973	0	1
168	0	1.16187	0	Infinite	5.26206	0.77029	-7.55748	0.802657	0.3703
169	0	1.64389	0	Infinite	7.44512	0.885238	-7.21678	1.19958	0.273406
170	0	0	0	Undefined	0	5.55E-17	-5.62162	0	1
171	2.78E-17	0	0	0	-1.26E-16	0	-3.91262	0	1
172	3.46287	0	0	0	-15.6832	0.936971	-7.22602	2.5379	0.111142
**173**	**5.13403**	**0**	**0**	**0**	**-23.2518**	**0.89147**	**-7.26866**	**3.37702**	**0.066111**

Negatively selected sites are highlighted in yellow and their corresponding significance values are highlighted in red.

Positively selected sites are highlighted in purple and their corresponding signficance value are shown in orange text

Table S5: Identification of Codon Sites Under Negative Selection: REL Estimates

Codon	E[dS]	E[dN]	E[dN-dS]	Posterior Pr{dN>dS}	Bayes Factor {dN>dS}	Posterior Pr{dN<dS}	Bayes Factor {dN<dS}
1	0.99997	0.144326	-0.85564	0.0035223	0.079322	0.996478	12.6069
2	0.798265	0.158794	-0.63947	0.0118485	0.269075	0.988152	3.71643
3	1.44766	0.159005	-1.28865	0.0074688	0.168866	0.992531	5.92186
4	0.796503	0.156496	-0.64001	0.0105918	0.240231	0.989408	4.16266
5	0.796498	0.151508	-0.64499	0.0078501	0.177554	0.99215	5.6321
6	0.796498	0.151508	-0.64499	0.0078501	0.177554	0.99215	5.6321
7	0.671066	0.152932	-0.51814	0.0092561	0.209653	0.990744	4.76979
8	1.57288	0.285063	-1.28782	0.0441544	1.03662	0.955846	0.964672
9	0.670978	0.159617	-0.51136	0.0132086	0.300378	0.986791	3.32914
10	0.67106	0.155799	-0.51526	0.0109474	0.248385	0.989053	4.02602
**11**	**2.36493**	**0.152555**	**-2.21237**	**0.0008894**	**0.019978**	**0.999111**	**50.0564**
12	0.798263	0.152493	-0.64577	0.0083844	0.189743	0.991616	5.2703
13	0.683683	0.155188	-0.5285	0.0105177	0.238532	0.989482	4.1923
14	0.705071	0.157129	-0.54794	0.0115221	0.261576	0.988478	3.82298
15	2.18517	1.08428	-1.1009	0.10551	2.64698	0.89449	0.377789
16	0.796503	0.156496	-0.64001	0.0105918	0.240231	0.989408	4.16266
17	0.683683	0.155188	-0.5285	0.0105177	0.238532	0.989482	4.1923
18	1.04623	0.168952	-0.87728	0.0149119	0.339698	0.985088	2.94379
19	0.796498	0.147347	-0.64915	0.0055959	0.126282	0.994404	7.91881
20	1.51642	0.151663	-1.36476	0.0046459	0.104744	0.995354	9.54712
21	0.594129	0.170297	-0.42383	0.0203979	0.467273	0.979602	2.14008
22	0.798265	0.158794	-0.63947	0.0118485	0.269075	0.988152	3.71643
23	1.46687	0.149901	-1.31697	0.0042365	0.095474	0.995764	10.474
24	0.708443	0.334194	-0.37425	0.114134	2.89122	0.885866	0.345875
25	2.27333	0.155193	-2.11814	0.0014961	0.033623	0.998504	29.7416
26	2.03622	0.169082	-1.86714	0.0048725	0.109877	0.995128	9.10107
27	1.45165	0.241853	-1.2098	0.0347128	0.806989	0.965287	1.23917
28	0.671017	0.151201	-0.51982	0.0082411	0.186472	0.991759	5.36275
29	0.745546	0.322101	-0.42345	0.104438	2.61695	0.895562	0.382124
30	0.67106	0.1499	-0.52116	0.0074858	0.169252	0.992514	5.90835
31	0.602556	0.16629	-0.43627	0.0178258	0.407282	0.982174	2.4553
32	0.683683	0.155188	-0.5285	0.0105177	0.238532	0.989482	4.1923
33	1.71541	0.252827	-1.46258	0.0293005	0.677369	0.970699	1.4763
34	0.798258	0.166355	-0.6319	0.0160335	0.365664	0.983967	2.73475
35	0.796498	0.151508	-0.64499	0.0078501	0.177554	0.99215	5.6321
36	0.796498	0.151508	-0.64499	0.0078501	0.177554	0.99215	5.6321
37	0.796503	0.156496	-0.64001	0.0105918	0.240231	0.989408	4.16266
38	1.28742	0.151207	-1.13621	0.0054499	0.12297	0.99455	8.13207
39	0.927753	0.169136	-0.75862	0.0163248	0.372418	0.983675	2.68516
40	0.671061	0.285197	-0.38586	0.0867994	2.13297	0.913201	0.46883
41	0.796503	0.156496	-0.64001	0.0105918	0.240231	0.989408	4.16266
42	0.7965	0.150838	-0.64566	0.0074891	0.169327	0.992511	5.90573
43	0.798265	0.158794	-0.63947	0.0118485	0.269075	0.988152	3.71643
44	0.796499	0.157155	-0.63934	0.0109519	0.248489	0.989048	4.02432

45	0.477209	0.424042	-0.05317	0.189609	5.25049	0.810391	0.190458
46	1.71197	0.152509	-1.55946	0.003952	0.089038	0.996048	11.2312
47	0.47654	0.169	-0.30754	0.0208143	0.477015	0.979186	2.09637
48	0.79649	0.345996	-0.45049	0.115337	2.92567	0.884663	0.341802
49	0.798258	0.166355	-0.6319	0.0160335	0.365664	0.983967	2.73475
50	1.28423	0.392418	-0.89181	0.100609	2.51029	0.899391	0.39836
51	1.56161	0.149944	-1.41166	0.0039165	0.088233	0.996084	11.3336
52	0.798258	0.166355	-0.6319	0.0160335	0.365664	0.983967	2.73475
53	2.21557	0.166324	-2.04925	0.0029139	0.065582	0.997086	15.2482
54	0.676387	0.285292	-0.3911	0.0865861	2.12724	0.913414	0.470094
55	0.843176	0.152484	-0.69069	0.0081648	0.184731	0.991835	5.41327
56	1.70839	0.158959	-1.54943	0.0056661	0.127875	0.994334	7.82011
57	0.707969	0.457341	-0.25063	0.188544	5.21413	0.811456	0.191786
58	2.25172	0.152573	-2.09914	0.0013957	0.031365	0.998604	31.8831
59	0.571054	0.169003	-0.40205	0.0198302	0.454004	0.98017	2.20262
60	0.999985	0.160583	-0.8394	0.0113358	0.257298	0.988664	3.88654
61	0.683675	0.147567	-0.53611	0.0060875	0.137443	0.993913	7.27574
62	0.796503	0.156496	-0.64001	0.0105918	0.240231	0.989408	4.16266
63	0.670978	0.159617	-0.51136	0.0132086	0.300378	0.986791	3.32914
64	0.99997	0.144326	-0.85564	0.0035223	0.079322	0.996478	12.6069
65	1.60741	0.929102	-0.67831	0.23479	6.88549	0.76521	0.145233
66	2.13386	0.1499	-1.98396	0.001562	0.035107	0.998438	28.4845
67	0.683681	0.1499	-0.53378	0.0074346	0.168087	0.992565	5.9493
68	0.738973	0.150108	-0.58887	0.0073269	0.165634	0.992673	6.03741
69	0.639921	0.147331	-0.49259	0.0060969	0.137658	0.993903	7.26439
70	0.796503	0.156496	-0.64001	0.0105918	0.240231	0.989408	4.16266
71	0.639924	0.156466	-0.48346	0.0115403	0.261995	0.98846	3.81687
**72**	**0.543562**	**1.48444**	**0.940881**	**0.877558**	**160.834**	**0.122442**	**0.006218**
73	0.798263	0.152493	-0.64577	0.0083844	0.189743	0.991616	5.2703
74	0.602558	0.152463	-0.4501	0.0093248	0.211224	0.990675	4.73431
75	0.556937	0.152554	-0.40438	0.0096056	0.217645	0.990394	4.59463
76	0.798265	0.158794	-0.63947	0.0118485	0.269075	0.988152	3.71643
77	0.609446	0.345873	-0.26357	0.127855	3.28975	0.872145	0.303975
78	0.705071	0.157129	-0.54794	0.0115221	0.261576	0.988478	3.82298
79	1.22753	0.169267	-1.05826	0.0131934	0.300027	0.986807	3.33303
80	0.798265	0.158794	-0.63947	0.0118485	0.269075	0.988152	3.71643
81	1.66222	0.159003	-1.50322	0.0060159	0.135817	0.993984	7.36285
82	0.684532	0.248359	-0.43617	0.0639921	1.5342	0.936008	0.651806
83	1.35569	0.157198	-1.19849	0.0073805	0.166855	0.992619	5.99323
84	0.7965	0.150838	-0.64566	0.0074891	0.169327	0.992511	5.90573
85	2.26255	0.155802	-2.10675	0.0016008	0.035981	0.998399	27.7923
**86**	**0.563424**	**1.53717**	**0.973749**	**0.903468**	**210.028**	**0.0965318**	**0.004761**
87	2.23795	0.413969	-1.82398	0.0266468	0.61434	0.973353	1.62776
88	0.556936	0.15519	-0.40175	0.0112553	0.255451	0.988745	3.91465
89	0.796503	0.156496	-0.64001	0.0105918	0.240231	0.989408	4.16266
90	0.63991	0.256237	-0.38367	0.0704233	1.70007	0.929577	0.588213
**91**	**2.53836**	**0.159559**	**-2.3788**	**0.0002406**	**0.0054**	**0.999759**	**185.173**

92	0.639921	0.157124	-0.4828	0.0119324	0.271004	0.988068	3.68998
93	0.594129	0.170297	-0.42383	0.0203979	0.467273	0.979602	2.14008
94	0.796503	0.156496	-0.64001	0.0105918	0.240231	0.989408	4.16266
95	0.738973	0.150108	-0.58887	0.0073269	0.165634	0.992673	6.03741
96	2.25694	0.155199	-2.10174	0.001587	0.035669	0.998413	28.0358
97	0.67106	0.155799	-0.51526	0.0109474	0.248385	0.989053	4.02602
98	0.594129	0.170297	-0.42383	0.0203979	0.467273	0.979602	2.14008
99	1.50271	0.160626	-1.34208	0.0075552	0.170835	0.992445	5.85361
100	0.683681	0.1499	-0.53378	0.0074346	0.168087	0.992565	5.9493
101	1.5019	0.149901	-1.352	0.0040996	0.092377	0.9959	10.8252
102	1.33085	0.157303	-1.17355	0.0077676	0.175674	0.992232	5.69235
103	0.798263	0.152493	-0.64577	0.0083844	0.189743	0.991616	5.2703
104	1.4817	0.147542	-1.33416	0.0034131	0.076854	0.996587	13.0117
105	0.780521	1.0244	0.243876	0.504545	22.8523	0.495455	0.043759
106	2.31653	0.1499	-2.16663	0.0009036	0.020296	0.999096	49.2706
107	0.796501	0.15956	-0.63694	0.0122784	0.278961	0.987722	3.58473
108	0.798258	0.166355	-0.6319	0.0160335	0.365664	0.983967	2.73475
109	0.671061	0.15519	-0.51587	0.0105923	0.240243	0.989408	4.16245
**110**	**2.53099**	**0.149899**	**-2.38109**	**0.0001541**	**0.003459**	**0.999846**	**289.135**
111	1.69687	0.15651	-1.54036	0.0050697	0.114347	0.99493	8.74529
112	0.639922	0.15956	-0.48036	0.0133995	0.304777	0.9866	3.28109
113	0.556908	0.159616	-0.39729	0.0140349	0.319435	0.985965	3.13052
114	0.999985	0.160583	-0.8394	0.0113358	0.257298	0.988664	3.88654
115	0.738973	0.150108	-0.58887	0.0073269	0.165634	0.992673	6.03741
116	0.798265	0.158794	-0.63947	0.0118485	0.269075	0.988152	3.71643
117	0.639922	0.15956	-0.48036	0.0133995	0.304777	0.9866	3.28109
118	0.671061	0.15519	-0.51587	0.0105923	0.240243	0.989408	4.16245
119	1.44114	0.151774	-1.28936	0.0050505	0.113912	0.994949	8.77871
120	0.801465	0.278238	-0.52323	0.0767403	1.86524	0.92326	0.536125
**121**	**2.50755**	**0.155799**	**-2.35175**	**0.0003063**	**0.006876**	**0.999694**	**145.428**
122	0.796503	0.156496	-0.64001	0.0105918	0.240231	0.989408	4.16266
123	0.796498	0.151508	-0.64499	0.0078501	0.177554	0.99215	5.6321
124	0.798265	0.158794	-0.63947	0.0118485	0.269075	0.988152	3.71643
125	0.7965	0.150838	-0.64566	0.0074891	0.169327	0.992511	5.90573
126	0.639924	0.156466	-0.48346	0.0115403	0.261995	0.98846	3.81687
127	0.796501	0.15956	-0.63694	0.0122784	0.278961	0.987722	3.58473
128	0.671065	0.146697	-0.52437	0.0056267	0.12698	0.994373	7.87523
129	0.7965	0.150838	-0.64566	0.0074891	0.169327	0.992511	5.90573
130	0.683665	0.158349	-0.52532	0.0123717	0.281107	0.987628	3.55736
131	0.67106	0.155799	-0.51526	0.0109474	0.248385	0.989053	4.02602
132	0.579008	0.15712	-0.42189	0.0123257	0.280049	0.987674	3.5708
133	0.671066	0.152932	-0.51814	0.0092561	0.209653	0.990744	4.76979
134	0.670978	0.159617	-0.51136	0.0132086	0.300378	0.986791	3.32914
135	0.670978	0.159617	-0.51136	0.0132086	0.300378	0.986791	3.32914
136	0.798265	0.158794	-0.63947	0.0118485	0.269075	0.988152	3.71643
137	1.3572	0.159158	-1.19804	0.0081781	0.185034	0.991822	5.40442
138	0.798258	0.166355	-0.6319	0.0160335	0.365664	0.983967	2.73475

139	0.923034	0.16902	-0.75401	0.0161399	0.368132	0.98386	2.71642
140	0.683681	0.1499	-0.53378	0.0074346	0.168087	0.992565	5.9493
141	0.796503	0.156496	-0.64001	0.0105918	0.240231	0.989408	4.16266
142	0.683678	0.155809	-0.52787	0.010879	0.246817	0.989121	4.05158
143	0.556936	0.1499	-0.40704	0.0079543	0.17993	0.992046	5.5577
144	0.639921	0.151486	-0.48844	0.0085525	0.193578	0.991448	5.16586
145	0.601884	0.347823	-0.25406	0.1296	3.34133	0.8704	0.299282
146	0.7965	0.150838	-0.64566	0.0074891	0.169327	0.992511	5.90573
147	0.60256	0.158751	-0.44381	0.0131765	0.299636	0.986824	3.33738
148	1.67034	0.152704	-1.51764	0.0042588	0.095979	0.995741	10.419
149	0.798265	0.158794	-0.63947	0.0118485	0.269075	0.988152	3.71643
150	0.816287	0.345457	-0.47083	0.113709	2.87909	0.886291	0.347332
151	0.798295	0.408085	-0.39021	0.150415	3.973	0.849585	0.251699
152	1.28577	1.23613	-0.04963	0.481934	20.8755	0.518066	0.047903
153	1.20134	0.968484	-0.23286	0.360611	12.6563	0.639389	0.079012
154	1.18329	0.279304	-0.90399	0.0596718	1.42405	0.940328	0.702224
155	1.34163	0.173715	-1.16792	0.0136901	0.311479	0.98631	3.21049
**156**	**2.47674**	**0.160445**	**-2.31629**	**0.0006178**	**0.013873**	**0.999382**	**72.0845**
157	0.59914	0.299023	-0.30012	0.0989161	2.46341	0.901084	0.405941
158	0.794747	0.335031	-0.45972	0.109363	2.75553	0.890637	0.362907
159	0.999968	0.146521	-0.85345	0.0045554	0.102693	0.995445	9.73776
160	0.816266	0.169817	-0.64645	0.0177715	0.406019	0.982229	2.46294
161	1.72827	0.155549	-1.57272	0.0046471	0.104772	0.995353	9.54457
162	0.816271	0.15552	-0.66075	0.0099387	0.225269	0.990061	4.43913
163	0.816273	0.162137	-0.65414	0.0135531	0.308318	0.986447	3.24341
164	0.835426	0.327101	-0.50833	0.102239	2.55558	0.897761	0.3913
165	0.708211	0.304311	-0.4039	0.0964099	2.39434	0.90359	0.417652
166	0.628985	0.169751	-0.45923	0.019692	0.450776	0.980308	2.21839
167	0.665761	0.28155	-0.38421	0.0848332	2.08018	0.915167	0.480728
168	0.624083	0.366775	-0.25731	0.139907	3.65031	0.860093	0.27395
169	0.665776	0.150667	-0.51511	0.007952	0.179878	0.992048	5.55933
170	0.695941	0.162978	-0.53296	0.0149993	0.341719	0.985001	2.92638
171	1.59077	0.1556	-1.43517	0.0054317	0.122556	0.994568	8.15955
172	1.70618	0.150738	-1.55544	0.0035319	0.079538	0.996468	12.5726

Table S6: Identification of Codon Sites Under Episodic Positive Selection: MEME Estimates

Codon	alpha	beta1	p1	beta2	p2	LRT	p-value	q-value	Log(L)
1	0	0	0	0	1	0	1	1	0
2	0	0	0	0	1	0	1	1	0
3	2.3279	0	0.04189	0	0.95811	0	0.67	1	-6.28439
4	0	0	0	0	1	0	1	1	0
5	0	0	0	0	1	0	1	1	0
6	0	0	0	0	1	0	1	1	0
7	0	0	0	0	1	0	1	1	0
8	3.21121	0.78662	0.995478	1.79577	0.004522	0	0.67	1	-14.0574
9	0	0	0	0	1	0	1	1	0
10	0	0	0	0	1	0	1	1	0
11	7.48075	0	0.047376	0	0.952625	0	0.67	1	-10.2645
12	0	0	0	0	1	0	1	1	0
13	0	0	0	0	1	0	1	1	0
14	0	0	0	0	1	0	1	1	0
15	4.01322	2.20702	1	2.20274	1.00E-09	0	0.67	1	-18.7011
16	0	0	0	0	1	0	1	1	0
17	0	0	0	0	1	0	1	1	0
18	1.23913	0	0.046772	0	0.953228	0	0.67	1	-8.20415
19	0	0	0	0	1	0	1	1	0
20	2.70329	0	0.047417	0	0.952583	0	0.67	1	-7.61585
21	0	0	0	0	1	0	1	1	0
22	0	0	0	0	1	0	1	1	0
23	2.38763	0	0.077281	0	0.922719	0	0.67	1	-7.0545
24	0	0	1.00E-09	0.966313	1	0.622783	0.399549	1	-8.5968
25	6.05549	0	0.047423	0	0.952577	0	0.67	1	-12.5844
26	2.98379	0	0.04732	0	0.95268	0	0.67	1	-12.6577
27	2.29123	0	0.941867	58.0801	0.058133	1.33017	0.2645	1	-15.2969
28	0	0	0	0	1	0	1	1	0
29	0	0	1.00E-09	0.929548	1	0.523624	0.425112	1	-7.87372
30	0	0	0	0	1	0	1	1	0
31	0	0	0	0	1	0	1	1	0
32	0	0	0	0	1	0	1	1	0
33	9.69362	0	0.901679	15.6637	0.098321	0.051047	0.606821	1	-15.4158
34	0	0	0	0	1	0	1	1	0
35	0	0	0	0	1	0	1	1	0
36	0	0	0	0	1	0	1	1	0
37	0	0	0	0	1	0	1	1	0
38	1.73776	0	0.047303	0	0.952698	0	0.67	1	-8.30081
39	1.07468	0	0.046586	0	0.953414	0	0.67	1	-8.33934
40	0	0	0.914206	23.2798	0.085794	1.36615	0.259222	1	-9.47782
41	0	0	0	0	1	0	1	1	0
42	0	0	0	0	1	0	1	1	0
43	0	0	0	0	1	0	1	1	0
44	0	0	0	0	1	0	1	1	0
45	0	0	0.753897	6.35692	0.246103	1.97417	0.185568	1	-10.05
46	7.4614	0	0.047476	0	0.952524	0	0.67	1	-8.03905
47	0	0	0	0	1	0	1	1	0

48	0	0	1.00E-09	1.01599	1	0.449963	0.445718	1	-8.32859
49	0	0	0	0	1	0	1	1	0
50	1.78046	1.23027	1	1.22772	1.00E-09	0	0.67	1	-14.2937
51	2.89865	0	0.047337	0	0.952664	0	0.67	1	-7.11276
52	0	0	0	0	1	0	1	1	0
53	4.68776	0	0.047392	0	0.952608	0	0.67	1	-12.0581
54	0	0	0.904061	19.278	0.095939	1.26363	0.274585	1	-9.5429
55	0	0	0	0	1	0	1	1	0
56	3.87373	0	0.047879	0	0.952121	0	0.67	1	-6.19571
57	0	0	1.00E-09	1.61303	1	0.983569	0.322228	1	-7.84697
58	5.40028	0	0.046671	0	0.953329	0	0.67	1	-9.63539
59	0	0	0	0	1	0	1	1	0
60	0	0	0	0	1	0	1	1	0
61	0	0	0	0	1	0	1	1	0
62	0	0	0	0	1	0	1	1	0
63	0	0	0	0	1	0	1	1	0
64	0	0	0	0	1	0	1	1	0
65	2.97707	0	0.854607	38.6442	0.145393	2.0505	0.178059	1	-17.0947
66	3.536	0	0.04735	0	0.95265	0	0.67	1	-11.1582
67	0	0	0	0	1	0	1	1	0
68	0	0	0	0	1	0	1	1	0
69	0	0	0	0	1	0	1	1	0
70	0	0	0	0	1	0	1	1	0
71	0	0	0	0	1	0	1	1	0
**72**	**0**	**0**	**1.00E-09**	**3.666**	**1**	**3.70834**	**0.074024**	**1**	**-18.6269**
73	0	0	0	0	1	0	1	1	0
74	0	0	0	0	1	0	1	1	0
75	0	0	0	0	1	0	1	1	0
76	0	0	0	0	1	0	1	1	0
77	0	0	0.902458	25.8172	0.097542	1.59153	0.228723	1	-10.1173
78	0	0	0	0	1	0	1	1	0
79	1.60504	0	0.047297	0	0.952703	0	0.67	1	-8.52702
80	0	0	0	0	1	0	1	1	0
81	3.87041	0	0.047851	0	0.952149	0	0.67	1	-5.75009
82	0	0	0.899639	14.4981	0.100361	1.09263	0.302601	1	-9.66176
83	1.94489	0	0.049947	0	0.950053	0	0.67	1	-7.0381
84	0	0	0	0	1	0	1	1	0
85	5.5791	0	0.050005	0	0.949995	0	0.67	1	-13.1426
**86**	**0**	**0**	**0.291295**	**5.22959**	**0.708705**	**3.93248**	**0.065863**	**1**	**-17.0724**
87	4.74802	1.34313	1	1.34291	1.00E-09	0	0.67	1	-12.0906
88	0	0	0	0	1	0	1	1	0
89	0	0	0	0	1	0	1	1	0
90	0	0	0.088536	0.773881	0.911465	0.592405	0.407139	1	-8.8432
91	14.2499	0	0.047314	0	0.952686	0	0.67	1	-11.9326
92	0	0	0	0	1	0	1	1	0
93	0	0	0	0	1	0	1	1	0
94	0	0	0	0	1	0	1	1	0
95	0	0	0	0	1	0	1	1	0
96	5.11936	0	0.047296	0	0.952704	0	0.67	1	-10.3149

97	0	0	0	0	1	0	1	1	0
98	0	0	0	0	1	0	1	1	0
99	2.63458	0	0.077331	0	0.922669	0	0.67	1	-7.40342
100	0	0	0	0	1	0	1	1	0
101	2.64927	0	0.05809	0	0.94191	0	0.67	1	-8.6323
102	1.88743	0	0.047417	0	0.952583	0	0.67	1	-8.77436
103	0	0	0	0	1	0	1	1	0
104	2.56328	0	0.048016	0	0.951984	0	0.67	1	-10.6553
105	0	0	1.00E-09	1.99373	1	0.9304	0.332351	1	-10.2425
106	7.66477	0	0.047274	0	0.952726	0	0.67	1	-10.3292
107	0	0	0	0	1	0	1	1	0
108	0	0	0	0	1	0	1	1	0
109	0	0	0	0	1	0	1	1	0
110	12.2005	0	0.046673	0	0.953327	0	0.67	1	-12.7288
111	5.78237	0	0.047993	0	0.952007	0	0.67	1	-8.58447
112	0	0	0	0	1	0	1	1	0
113	0	0	0	0	1	0	1	1	0
114	0	0	0	0	1	0	1	1	0
115	0	0	0	0	1	0	1	1	0
116	0	0	0	0	1	0	1	1	0
117	0	0	0	0	1	0	1	1	0
118	0	0	0	0	1	0	1	1	0
119	2.28992	0	0.050031	0	0.949969	0	0.67	1	-7.2842
120	0	0	0.901223	17.8454	0.098777	0.98596	0.321782	1	-10.9045
121	8.62123	0	0.046586	0	0.953414	0	0.67	1	-17.1279
122	0	0	0	0	1	0	1	1	0
123	0	0	0	0	1	0	1	1	0
124	0	0	0	0	1	0	1	1	0
125	0	0	0	0	1	0	1	1	0
126	0	0	0	0	1	0	1	1	0
127	0	0	0	0	1	0	1	1	0
128	0	0	0	0	1	0	1	1	0
129	0	0	0	0	1	0	1	1	0
130	0	0	0	0	1	0	1	1	0
131	0	0	0	0	1	0	1	1	0
132	0	0	0	0	1	0	1	1	0
133	0	0	0	0	1	0	1	1	0
134	0	0	0	0	1	0	1	1	0
135	0	0	0	0	1	0	1	1	0
136	0	0	0	0	1	0	1	1	0
137	1.96562	0	0.054761	0	0.945239	0	0.67	1	-5.95684
138	0	0	0	0	1	0	1	1	0
139	1.06938	0	0.047328	0	0.952672	0	0.67	1	-7.49902
140	0	0	0	0	1	0	1	1	0
141	0	0	0	0	1	0	1	1	0
142	0	0	0	0	1	0	1	1	0
143	0	0	0	0	1	0	1	1	0
144	0	0	0	0	1	0	1	1	0
145	0	0	0.935246	76.1862	0.064754	2.46954	0.142193	1	-10.6232

146	0	0	0	0	1	0	1	1	0
147	0	0	0	0	1	0	1	1	0
148	4.23389	0	0.047427	0	0.952573	0	0.67	1	-5.73072
149	0	0	0	0	1	0	1	1	0
150	0	0	0.027787	1.06945	0.972213	0.407505	0.458329	1	-8.9624
151	0	0	0.100857	1.49519	0.899143	0.557666	0.416075	1	-8.33303
**152**	**0**	**0**	**0.950577**	**353.352**	**0.049423**	**8.0966**	**0.007768**	**0.668064**	**-11.7858**
**153**	**0**	**0**	**0.95289**	**312.981**	**0.047111**	**14.8307**	**0.000257**	**0.044251**	**-14.2302**
154	1.49126	0.753575	0.990375	2.50251	0.009625	6.53E-05	0.668045	1	-14.5944
155	1.93054	0	0.046687	0	0.953313	0	0.67	1	-9.6055
156	9.1964	0	0.047296	0	0.952704	0	0.67	1	-10.3169
157	0	0	1.00E-09	0.833877	1	0.802932	0.358272	1	-7.7722
158	0	0	0.886087	16.2038	0.113913	0.963018	0.326096	1	-9.42636
159	0.844619	0	0.046586	0	0.953414	0	0.67	1	-4.08746
160	0	0	0.046825	0	0.953175	0	0.67	1	-3.32328
161	10.2398	0	0.047986	0	0.952014	0	0.67	1	-7.57236
162	0	0	0.046825	0	0.953175	0	0.67	1	-3.30648
163	0	0	0.046825	0	0.953175	0	0.67	1	-2.81415
164	0	0	1.00E-09	0.937263	1	0.327901	0.483696	1	-6.97651
165	0	0	1.00E-09	0.851806	1	0.566478	0.413782	1	-7.19398
166	0	0	0.046825	0	0.953175	0	0.67	1	-4.15973
167	0	0	1.00E-09	0.774194	1	0.601255	0.404907	1	-7.85011
168	0	0	1.00E-09	1.09912	1	0.925217	0.333359	1	-7.50224
169	0	0	0.046825	0	0.953175	0	0.67	1	-5.62162
170	0	0	0.046825	0	0.953175	0	0.67	1	-3.91262
171	3.30608	0	0.047296	0	0.952704	0	0.67	1	-7.171
172	4.82763	0	0.047325	0	0.952675	0	0.67	1	-7.23107

Table S7. GA Branch analysis for predicting lineages subject to positive selection in a phylogeny.

Branch Name	Mean	Std.Dev.	2.50%	Median	97.50%	Prob{dN>dS}
**ANKB1**	**1.28856**	**0.161651**	**0.891215**	**1.3522**	**1.55372**	**0.926624**
ANKB8	1.1892	91.5382	0	0.117254	1.38851	0.217019
**Node3**	**37.2876**	**599.053**	**0.670634**	**1.3522**	**1.5587**	**0.907126**
**ANKB3**	**37.4674**	**600.381**	**0.883912**	**1.3522**	**1.5587**	**0.923469**
Node2	0.026545	0.059725	0	0	0.140263	3.73E-06
ANKB2	0.178494	0.169778	0.114442	0.144928	1.03761	0.025866
**ANKB9**	**13.0434**	**344.366**	**0.137317**	**1.3492**	**1.55322**	**0.831843**
**Node7**	**1.24666**	**0.252218**	**0.155972**	**1.3522**	**1.55322**	**0.894997**
Node1	0.035724	0.085305	0	0	0.142726	0.000704
ANKB4	3.78139	180.987	0	0.143327	1.38855	0.335493
**ANKB11**	**1.24199**	**0.2588**	**0.156083**	**1.3522**	**1.54083**	**0.891616**
Node13	0.050479	0.120149	0	0	0.154413	0.007806
**ANKB10**	**87.778**	**925.892**	**0.889259**	**1.3522**	**1.55876**	**0.927625**
Node12	2.13885	132.747	0	0.137137	1.38849	0.235613
ANKB7	0.050005	0.13202	0	0	0.151416	0.010348
Node11	0.038147	0.10047	0	0	0.142788	0.005037
ANKB6	2.04916	128.308	0	0.145123	1.37204	0.227074
ANKB12	0.055069	0.128535	0	0	0.153279	0.008705
Node19	0.14472	0.011074	0.121889	0.144493	0.163694	0
ANKB13	19.5775	435.816	0	0.146566	1.51265	0.359103
ANKB14	0.215915	0.25391	0.114199	0.146566	1.14409	0.061191
ANKB15	0.129388	0.038563	0.023218	0.143994	0.156083	0
Node24	0.143052	0.015734	0.111103	0.144007	0.159941	0
Node22	0.142735	0.013147	0.109176	0.144006	0.157656	0
Node18	0.172242	0.134313	0.125299	0.145812	0.825389	0
Node10	0.192895	0.214477	0.112517	0.145768	1.16333	0.04243
ANKB5	0.151788	0.086251	0.10992	0.144447	0.166317	0.000344

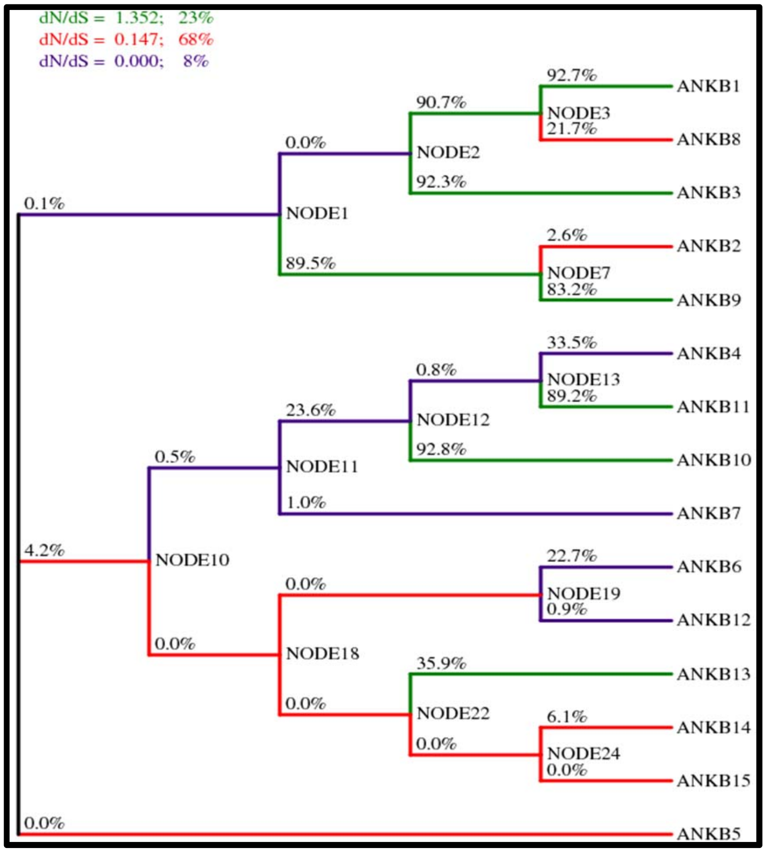
dN/dS = 1.352; 23%
dN/dS = 0.147; 68%
dN/dS = 0.000; 8%
92.7%
ANKB1
90.7%
NODE3
21.7%
ANKB8
0.0%
NODE2
92.3%
ANKB3
0.1%
NODE1
2.6%
ANKB2
89.5%
NODE7
83.2%
ANKB9
33.5%
ANKB4
0.8%
NODE13
89.2%
ANKB11
23.6%
NODE12
92.8%
ANKB10
0.5%
NODE11
1.0%
ANKB7
4.2%
NODE10
22.7%
ANKB6
0.0%
NODE19
0.9%
ANKB12
0.0%
NODE18
35.9%
ANKB13
0.0%
NODE22
6.1%
ANKB14
0.0%
NODE24
0.0%
ANKB15
0.0%
ANKB5

Legends to Supplementary Figures

Fig. S1. Sequence polymorphism and divergence in *ankB* alleles. Polymorphisms unique to each *ankB* allele are shown. Site numbers for each variable position is shown in a vertical format. *ankB2* allele sequence was used as a reference. This analysis was done using MEGA 6 (1).

Fig. S2. Two recombination breakpoints were identified in *ankB* alignment. Statistical support for each breakpoint is shown. Breakpoint at position 250 was also significant for a Kishino Hasegawa test indicating the presence of 2 or more incongruent topologies for *ankB* alignment. This analysis was done using GARD (5)

Fig. S3. *ankB* lineages have experienced variable selective pressures. An unscaled *ankB* phylogeny indicating the associated selective pressures for each branch is shown. This GA Branch analysis indicates a 92.7% and 90.7% support for positive selection on the *ankB1* branch and the internal node leading up to *ankB1*, respectively.

Fig. S4. Environmental and clinical isolates showed modest, but significant, difference in their ability to recruit polyubiquitinated proteins. Box-plots show % polyubqcuitination (ability to recruit polyubiquitinated proteins to the LCV) among 10 environmental and 22 clinical isolates. Statistical significance was analyzed via a two tailed Mann-Whitney nonparametric test. The Z-Score was 2.0111; the p-value is 0.04444. The critical value of U at $p \leq 0.05$ was 29. Therefore, the difference between clinical and environmental isolates is significant at $p \leq 0.05$.

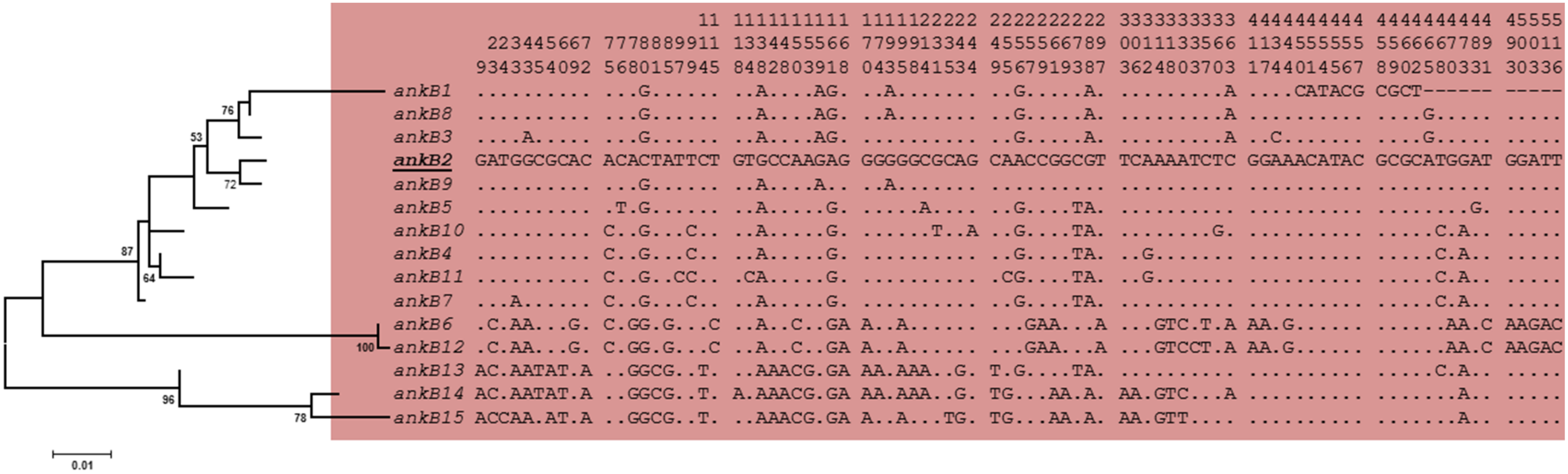
11 1111111111 1111122222 2222222222 3333333333 4444444444 4444444444 45555
223445667 7778889911 1334455566 7799913344 4555566789 0011133566 1134555555 5566667789 90011
9343354092 5680157945 8482803918 0435841534 9567919387 3624803703 1744014567 8902580331 30336
ankB1 ....G...... ..A....AG. ..A....... ..G.....A. ....A ....CATACG CGCT------ -----
ankB8 ....G...... ..A....AG. ..A....... ..G.....A. ....A ....G..... .....
ankB3 ....A..... ...G...... ..A....AG. ....G.....A. ....A ..C....... ....G..... .....
ankB2 GATGGCGCAC ACACTATTCT GTGCCAAGAG GGGGGCGCAG CAACCGGCGT TCAAAATCTC GGAAACATAC GCGCATGGAT GGATT
ankB9 ....G...... ..A....A.. ..A....... ....
ankB5 .... .T.G...... ..A.....G. .....A.... ..G....TA. ....G. .....
ankB10 .... C..G...C.. ..A.....G. ......T..A ..G....TA. ....G. ....C.A.. .....
ankB4 .... C..G...C.. ..A.....G. ....G....TA. ..G....... ....C.A.. .....
ankB11 .... C..G..CC.. .CA.....G. .... .CG....TA. ..G....... ....C.A.. .....
ankB7 ...A...... C..G...C.. ..A.....G. ....G....TA. ....C.A.. .....
ankB6 .C.AA...G. C.GG.G...C ..A..C..GA A..A...... ...GAA...A ...GTC.T.A AA.G...... ......AA.C AAGAC
ankB12 .C.AA...G. C.GG.G...C ..A..C..GA A..A...... ...GAA...A ...GTCCT.A AA.G...... ......AA.C AAGAC
ankB13 AC.AATAT.A ..GGCG..T. ..AAACG.GA AA.AAA..G. T.G....TA. ....C.A.. .....
ankB14 AC.AATAT.A ..GGCG..T. A.AAACG.GA AA.AAA..G. TG...AA.A. AA.GTC...A ....A.. .....
ankB15 ACCAA.AT.A ..GGCG..T. ..AAACG.GA A..A...TG. TG...AA.A. AA.GTT.... ....A.. .....
76
53
72
87
64
100
96
78
0.01

Figure S1

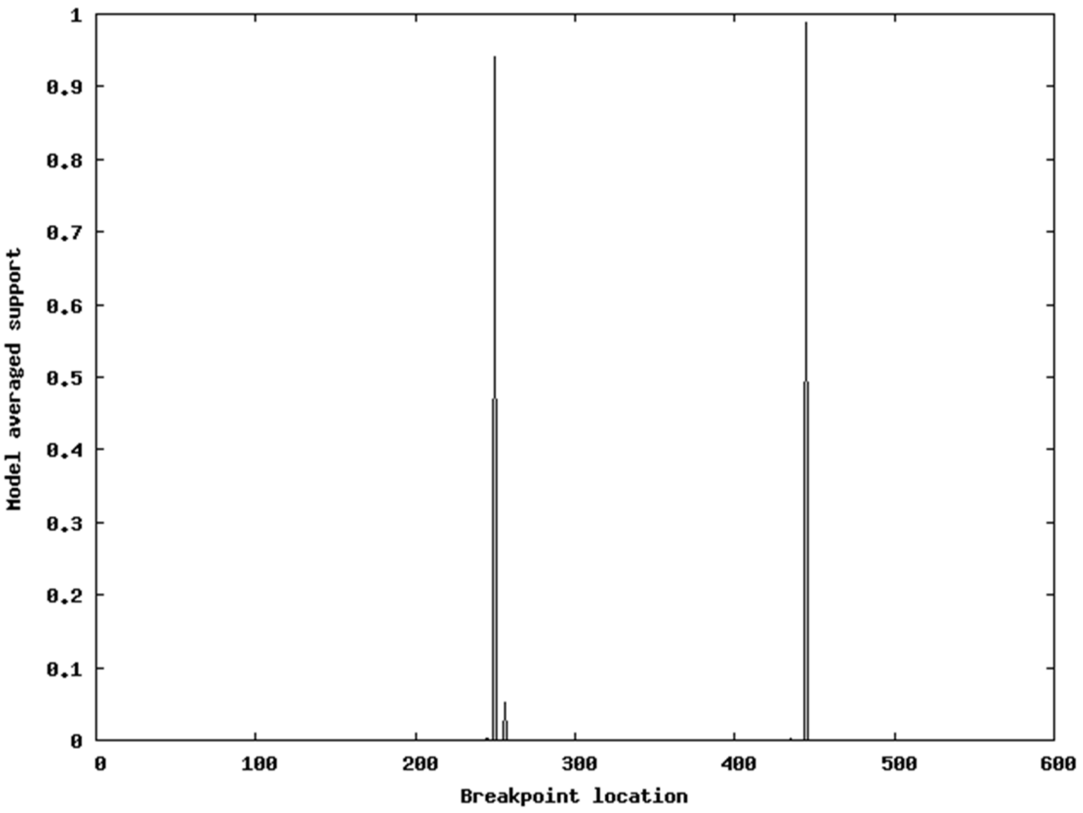

1
0.9
0.8
0.7
0.6
0.5
0.4
0.3
0.2
0.1
0
0
100
200
300
400
500
600
Model averaged support
Breakpoint location

Figure S2

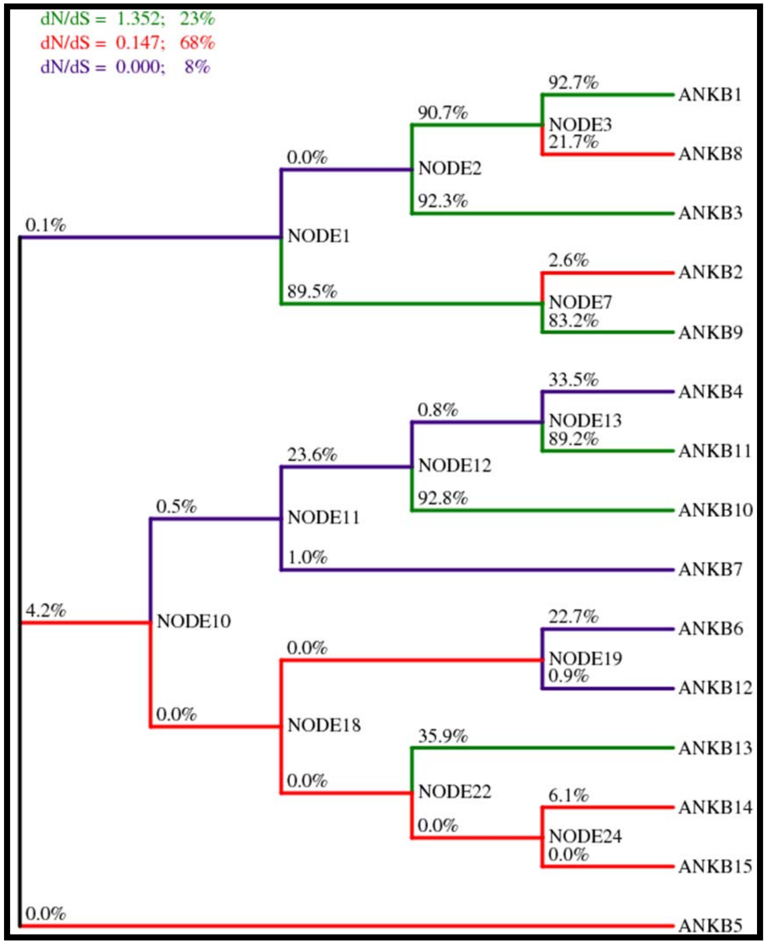
dN/dS = 1.352; 23%
dN/dS = 0.147; 68%
dN/dS = 0.000; 8%
92.7%
ANKB1
90.7%
NODE3
21.7%
ANKB8
0.0%
NODE2
92.3%
ANKB3
0.1%
NODE1
2.6%
ANKB2
89.5%
NODE7
83.2%
ANKB9
33.5%
ANKB4
0.8%
NODE13
89.2%
ANKB11
23.6%
NODE12
92.8%
ANKB10
0.5%
NODE11
1.0%
ANKB7
4.2%
NODE10
22.7%
ANKB6
0.0%
NODE19
0.9%
ANKB12
0.0%
NODE18
35.9%
ANKB13
0.0%
NODE22
6.1%
ANKB14
0.0%
NODE24
0.0%
ANKB15
0.0%
ANKB5

Figure S3

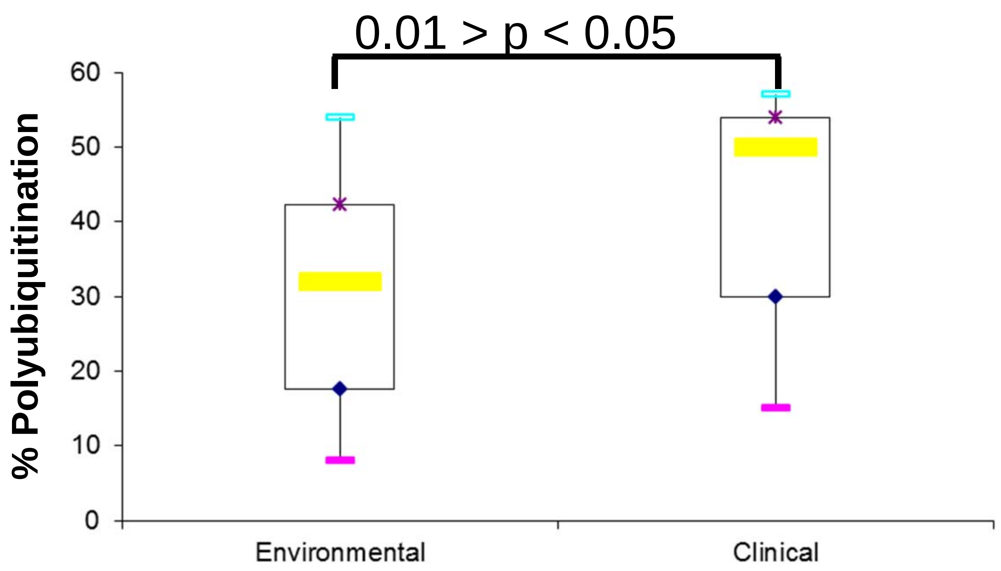
0.01 > p < 0.05
% Polyubiquitination
60
50
40
30
20
10
0
Environmental
Clinical

Figure S4
